# Bacterial wilt pathogen induced spatial shifts of root-associated microbiome and metabolome of potatoes

**DOI:** 10.3389/fpls.2025.1577123

**Published:** 2025-05-16

**Authors:** Xianjun Lai, Zhouhua He, Shuyan Wang, Feng Zhang, Haiyan Wang, Xiyao Wang, Shifeng Liu, Lang Yan

**Affiliations:** ^1^ Panxi Crops Research and Utilization Key Laboratory of Sichuan Province, College of Agriculture Science, Xichang University, Liangshan, China; ^2^ Potato Research and Development Center, College of Agriculture Science, Sichuan Agricultural University, Chengdu, China; ^3^ Sichuan Key Laboratory of Molecular Biology and Biotechnology, College of Life Sciences, Sichuan University, Chengdu, China

**Keywords:** bacterial wilt, root-associated microbiome, metabolome analysis, microbiome-metabolome interactions, potato

## Abstract

**Introduction:**

Plant root-associated microbiomes play an important role in plant health, yet their responses to bacterial wilt remain unclear poorly understood.

**Methods:**

This study investigated spatial variations in microbiome and metabolome composition across three root-associated niches—root-surrounding soil, rhizosphere, and endosphere—of healthy and *Ralstonia solanacearum*-infected potato plants. A total of 36 samples were analyzed, with microbial diversity assessed by full-length 16S rRNA and ITS sequencing, and metabolic profiles characterized using LC-QTOF-MS.

**Results:**

Alpha diversity analysis revealed that bacterial diversity in healthy plants was consistently higher than in diseased plants, progressively increasing from the root-surrounding soil to the rhizosphere, and most notably in the endosphere, where the Shannon index declined from 5.3 (healthy) to 1.2 (diseased). In contrast, fungal diversity was lower in diseased plants in the root-surrounding soil and rhizosphere, but significantly elevated in the endosphere, suggesting niche-specific microbial responses to pathogen stress. Beta diversity confirmed significant microbiome restructuring under pathogen stress (*R²* > 0.5, *p* = 0.001). Taxonomic analysis showed over 98% dominance of Proteobacteria in the diseased endosphere, where *Burkholderia*, *Pseudomonas*, and *Massilia* enriched in healthy plants were significantly reduced. *R. solanacearum* infection promotes the enrichment of *Fusarium* species in both the rhizosphere and endosphere. Metabolomic analysis revealed extensive pathogen-induced metabolic reprogramming, with 299 upregulated and 483 downregulated metabolites in the diseased endosphere, including antimicrobial metabolites such as verruculogen and aurachin A. Network analysis identified XTP as a central metabolite regulating microbial interactions, whereas antimicrobial metabolites exhibited targeted pathogen suppression. O2PLS analysis revealed that pathogen-induced antimicrobial metabolites (e.g., Gentamicin X2, Glutathionylspermine) were associated with *Clostridia* and *Ketobacter* in diseased plants, while nucleotide-related compounds (e.g., XTP) correlated with *Rhodomicrobium* and others, indicating infection-driven microbial adaptation and metabolic restructuring.

**Discussion:**

These findings provide insights into pathogen-driven disruptions in root microbiomes and suggest potential microbiome engineering strategies for bacterial wilt management.

## Introduction

In nature ecosystems, plants do not exist in isolation but interact with a highly diverse community of microorganisms, which form complex networks with their host plants and the surrounding environment (Maurice et al., 2024). Among these interactions, the plant root microbiome plays a pivotal role, mediating processes essential for plant health and development, such as nutrient acquisition, stress tolerance, and disease resistance (Trivedi et al., 2020; Tao et al., 2022). The assembly of these microbial communities is not random; instead, it is largely driven by host selection processes, wherein plants actively recruit beneficial microbes while excluding harmful ones (Attia et al., 2022). Root-associated microbiota is spatially structured, forming distinct niches across the rhizosphere (the soil influenced by root exudates), the root surface, and the endosphere (the root interior), each with unique microbial compositions and ecological functions (Bai et al., 2022; Ku et al., 2023). This spatial differentiation represents a continuum of microbial diversity transitioning from the bulk soil to the root interior, driven by the plant’s selective enrichment of beneficial microbes from the surrounding soil. These microbes thrive in the root-associated soil and are ultimately internalized by the plant to establish symbiotic or endophytic relationships (van der Heijden and Schlaeppi, 2015; Santos-Medellín et al., 2017). Microbial diversity and community composition within the root microbiome are closely associated with plant health, with high microbial diversity often correlated with robust microbial networks that enhance plant resilience to pathogens. Complex microbial interactions in such networks suppress pathogen growth through mechanisms including competition for resources, production of antimicrobial compounds, and induction of plant immune responses (Du et al., 2024). Notably, microbial diversity within the root microbiome decreases progressively from the rhizosphere to the endosphere, reflecting both environmental and host-imposed selection pressures (Dlamini et al., 2023). In this context, the rhizosphere acts as a dynamic interface and facilitates interactions between plants and both beneficial and pathogenic microbes, making it the first line of defense against soil-borne pathogens. In contrast, endophytic microbes establish more intimate associations with the host, directly influencing plant physiology and contributing to systemic resistance by promoting plant immune responses and mitigating pathogen-induced damage (Santoyo et al., 2016; Chiaramonte et al., 2021).

When plants are challenged by pathogens, they can actively alter their microbiome composition in a phenomenon known as the “cry for help hypothesis.” This hypothesis suggests that plants recruit beneficial microbes from the surrounding soil in response to pathogen attacks, reassembling the root microbiome to enhance their defenses (Li et al., 2022; Rolfe et al., 2019). The recruited microbes may directly inhibit pathogens through the production of antibiotics or siderophores or indirectly support plant health by modulating immune responses (Carrión et al., 2019). For example, *Stenotrophomonas rhizophila* (SR80) was enriched in the rhizosphere and endosphere of wheat infected with *Fusarium pseudograminearum* (Fp), and re-inoculation of SR80 in soil suppressed disease progression and enhanced plant growth (Liu et al., 2021); Similarly, *Arabidopsis thaliana* selectively promotes the growth of three rhizosphere bacteria to activate systemic defenses against the invasion of the downy mildew pathogen *Hyaloperonospora arabidopsis* (Berendsen et al., 2018); These examples underscore the adaptive capacity of plants to reshape their microbiomes for improved resilience under pathogen stress. Despite these advances, significant gaps remain in understanding how pathogen invasion drives spatial variations in microbiome composition across different niches. Additionally, the interplay between microbial community structure and metabolomic changes in response to pathogen stress is poorly characterized. Addressing these knowledge gaps will provide crucial insights into plant-microbiome interactions and inform strategies for sustainable disease management.

The bacterial wilt pathogen *Ralstonia solanacearum* (Rs) is a Gram-negative soil-borne bacterium that infects over 200 plant species, including economically significant crops such as potato, tomato, and eggplant (European and Mediterranean Plant Protection Organization, 2021). The invasion of Rs disrupts the root-associated microbiome, often triggering stress responses that reshape microbial community composition. Studies have reported that plant rhizosphere defenses against Rs invasion were mediated by bacterial resource competition and the recruitment of key beneficial microbes, supporting the positive diversity-invasion resistance relationship observed in healthy plant-associated microbial communities (Xiong et al., 2021; Van Elsas et al., 2012). For instance, previous study in peppers has identified beneficial microbes, including *Pseudomonas* and *Bacillus*, enriched in diseased plants (Gao et al., 2021).Similarly, some antibiotic-producing bacteria, including *Streptomyces*, *Bosea* and *Pseudomonas*, are enriched in tobacco with bacterial wilt, and serve as pathogen antagonists by producing antibiotics or outcompeting Rs for resources (Tao et al., 2022). We hypothesize that invading pathogen trigger stress responses in microbial communities directly or indirectly through plants, activating antagonistic traits and shifting in microbiome composition, thereby limiting pathogen invasion. After being attacked by pathogens, plants can use soil microbial communities to resist infection. Therefore, in the field with bacterial wilt outbreaks, although the environmental conditions of healthy and diseased plants are similar (attacked by pathogens), specific bacterial communities were hypothesized to be present in the root-associated niches of healthy plants to maintain the balance of microbial communities and help plants suppress the occurrence of diseases. However, most previous studies have only targeted structure changes of rhizosphere microbial communities, little is known about the ecological responses of endophytic communities and metabolites across different root-associated niches to bacterial wilt invasion.

To address these knowledge gap, our study investigates the spatial variations in microbiome and metabolome composition across three root-associated niches—the root surrounding soil, the rhizosphere and the endosphere—of healthy and Rs-infected potato plants. Root samples were collected from a bacterial wilt outbreak field, and microbiome diversity was analyzed using 16S rRNA and ITS sequencing, complemented by untargeted metabolomic profiling. Following the framework established by Donn et al (Donn et al., 2015), we classified the root microbiome into loosely associated (L compartment, mainly representing microorganisms living in root-surrounding soil and rhizosphere) and tightly associated (T compartment, mainly representing endophytes) communities to distinguish epiphytic and endophytic microbes, respectively. The objectives of this study are threefold: (i) to elucidate taxonomic and functional shifts in root-associated microbiomes under Rs stress, (ii) to identify key microbial taxa and metabolites that positively influence plant health under pathogen attack, and (iii) to compare the microbial networks and metabolomic profiles of healthy and diseased plants across different niches to gain insights into community stability. Our findings provide a detailed assessment of niche-specific responses to pathogen invasion, highlighting the diverse strategies employed by beneficial microbes to inhibit Rs. This study advances our understanding of microbial assembly mechanisms and defense strategies in plant-microbe interactions and offers potential applications for sustainable bacterial wilt management.

## Materials and methods

### Experimental design and sample collection

To investigate microbial diversity and community composition under bacterial wilt stress, samples were collected from healthy and diseased potato plants in a field experiencing an active bacterial wilt outbreak located at Butuo county, Liangshan (latitude 27.72°N and longitude 102.79°E, with an elevation of 2,385 meters above sea level). Sampling was conducted in June 2024, and root-associated soil compartments were defined based on their physical proximity to the root system, including root-surrounding soil (soil loosely shaken from roots), rhizosphere (soil tightly adhering to roots), and endosphere (internal root tissues). Six healthy and six diseased plants were selected, and samples were collected from three root-associated niches: root-surrounding soil, rhizosphere and endosphere (root interior). A total of 36 samples were collected, with 12 from each niche (6 healthy and 6 diseased).

Root-surrounding sample was collected by shaking off loosely adhered soil. Rhizosphere samples were collected by transferring the root systems into sterile 50 mL centrifuge tubes containing 20 mL of sterile 10 mM phosphate-buffered saline (PBS: 137 mM NaCl, 2.7 mM KCl, 10 mM Na_2_HPO_4_, 1.8 mM KH_2_PO_4_, pH 7.4). The tubes were placed on an orbital shaker at 120 rpm for 20 minutes at room temperature to dislodge the rhizosphere soil (Beckers et al., 2017). After shaking, roots were removed using sterile forceps, and the remaining suspension was centrifuged at 6,000 × g for 20 minutes at 4°C. The resulting pellet was collected as the rhizosphere soil fraction. To obtain endosphere samples, roots were surface-sterilized by sequential washing with sterile water (30 s), 70% ethanol (2 min), 2.5% sodium hypochlorite containing 0.1% Tween 80 (5 min), and 70% ethanol (30 s), followed by five rinses with sterile water (Beckers et al., 2017). Sterilized root tissues were then sectioned with a sterile scalpel and homogenized in phosphate-buffered saline using a tissue homogenizer under aseptic conditions. Homogenates were stored at −80 °C until DNA extraction.

### DNA extraction and sequencing

For rhizosphere and rhizoplane samples, total genomic DNA was extracted using the TGuide S96 Magnetic Soil/Stool DNA Kit (Tiangen Biotech, Beijing, China), following the manufacturer’s instructions. DNA quality and quantity were assessed using 1.8% agarose gel electrophoresis and a NanoDrop 2000 UV-Vis spectrophotometer (Thermo Scientific, Wilmington, USA). Full-length 16S rRNA genes were amplified using barcoded primer pairs 27F (5’-AGRGTTTGATYNTGGCTCAG-3’) and 1492R (5’-TASGGHTACCTTGTTASGACTT-3’). For fungal communities, the full-length ITS region was amplified using primers ITS1F (5’-CTTGGTCATTTAGAGGAAGTAA-3’) and ITS4R (5’-TCCTCCGCTTATTGATATGC-3’). PCR amplification was performed using KOD One PCR Master Mix (TOYOBO Life Science) under the following conditions: 95°C for 2 min, 25 cycles of 98°C for 10 s, 55°C for 30 s, and 72°C for 1 min 30 s, with a final extension at 72°C for 2 min. PCR products were purified using VAHTS DNA Clean Beads (Vazyme, Nanjing, China) and quantified using the Qubit dsDNA HS Assay Kit and Qubit 3.0 Fluorometer (Invitrogen, Thermo Fisher Scientific, Oregon, USA). Equimolar amplicons were pooled, and SMRTbell libraries were prepared using the SMRTbell Express Template Prep Kit 2.0 (Pacific Biosciences). Sequencing was performed on a PacBio Sequel II platform (Beijing Biomarker Technologies, Beijing, China).

For endosphere samples, DNA was extracted using the same method. The bacterial 16S rRNA V3–V4 region was amplified using primers 338F (5′-ACTCCTACGGGAGGCAGCA-3′) and 806R (5′-GGACTACHVGGGTWTCTAAT-3′), while the ITS1 region of fungi was amplified using primers ITS1F (5′-CTTGGTCATTTAGAGGAAGTAA-3′) and ITS2 (5′-GCTGCGTTCTTCATCGATGC-3′). All primers included Illumina adapter sequences for multiplexing. PCR was carried out in 20 μL reactions containing 5–50 ng DNA template, 0.3 μL of each primer (10 μM), 5 μL KOD FX Neo Buffer, 2 μL dNTPs (2 mM each), 0.2 μL KOD FX Neo polymerase, and nuclease-free water. The cycling conditions were: 95 °C for 5 min; 20 cycles of 95 °C for 30 s, 50 °C for 30 s, and 72 °C for 40 s; and a final extension at 72 °C for 7 min. PCR products were purified with the Omega DNA purification kit (Omega Inc., Norcross, GA, USA), quality-checked using the Qsep-400 system (BiOptic Inc., Taiwan), and sequenced using the Illumina NovaSeq 6000 platform (paired-end 2×250 bp; Beijing Biomarker Technologies, Beijing, China).

### Data processing and diversity analysis

Clean reads from PacBio sequencing were processed into amplicon sequence variants (ASVs) using DADA2 in QIIME2 (Callahan et al., 2016). Reads with less than two counts across all samples were filtered out. For Illumina sequencing, paired-end reads were merged, quality filtered, and processed to generate ASVs using DADA2 (v1.20.0). Taxonomic annotation for all ASVs was performed with the Naive Bayes classifier in QIIME2 (Bolyen et al., 2019), referencing the SILVA database (release 138.1) at a confidence threshold of 70% (Quast et al., 2012).

Alpha diversity (e.g., Shannon and Chao1 indices) was calculated using QIIME2 (version 2020.6) and visualized using the ggplot2 package in R (version 4.2.2) to assess species richness and evenness. Alpha rarefaction curves were generated using the QIIME diversity analysis workflow script core_diversity_analyses.py. The Kruskal–Wallis test was applied to compare alpha diversity among groups. When significant differences were found, pairwise comparisons were conducted using the Wilcoxon rank-sum test, and *p*-values were corrected for false discovery rate.

Beta diversity was analyzed based on Bray-Curtis distance and visualized by principal coordinate analysis (PCoA) using the capscale() function in the vegan package in R (model: capscale(log2(RA) ~ 1)). Permutational multivariate analysis of variance (PERMANOVA) was performed using the adonis() function with 999 permutations to test for differences in microbial community composition. Additionally, analysis of similarities (ANOSIM) was conducted using the anosim() function to further assess the degree of group separation, with significance set at *p* < 0.05 (Oksanen et al., 2013). Genus-level abundance bar plots, UPGMA clustering (Gronau and Moran, 2007), and heatmaps were used to further explore community differences. Statistical significance was tested using one-way ANOVA.

### Differential abundance and functional prediction

Differentially abundant taxa between healthy and diseased groups were identified using Linear Discriminant Analysis Effect Size (LEfSe, v1.1.1) (Segata et al., 2011). LEfSe analysis was conducted using the online Galaxy module (https://huttenhower.sph.harvard.edu/galaxy/), with relative abundances of bacterial taxa as input. Taxonomic features were filtered at a minimum relative abundance of 0.1% across all samples. The Kruskal-Wallis test was used to detect significant differences between groups, followed by pairwise Wilcoxon tests. A logarithmic LDA score threshold of 4.0 was applied to select discriminative features. Random Forest (RF) analysis was conducted using the R package randomForest with microbial taxonomic abundances at phylum, class, order, family, and genus levels as input (Liaw and Wiener, 2002). For model training, a 10-fold cross-validation approach was applied to evaluate the classification accuracy. The minimum cross-validation error was used to determine the optimal number of taxa for each root-associated niche. Feature importance scores were calculated, and taxa with the highest importance values were identified as key biomarkers. Functional prediction of bacterial communities was performed using FAPROTAX v1.2.6 (Louca et al., 2016), focusing on ecological roles such as chemoheterotrophy, nitrogen fixation, plant pathogenesis, and organic matter degradation. Fungal functional prediction was performed using FUNGuild (August 2021 release), which assigns fungal taxa to ecologically meaningful guilds based on three major trophic modes: pathotrophs, symbiotrophs, and saprotrophs (Nguyen et al., 2016). All statistical analyses were performed in R, using the wilcox.test() function with the default paired = FALSE parameter, and FDR correction was conducted using the p.adjust() function with the “fdr” method.

### Metabolite extraction and sample preparation

Metabolite extraction was performed using a modified methanol/acetonitrile/water (2:2:1, v/v/v) protocol. Briefly, 50 mg of soil samples were mixed with 1000 μL of extraction solution containing 2 μL of L-2-chlorophenylalanine (internal standard, Aladdin, China) and vortexed. The mixture was homogenized with ceramic beads at 45 Hz for 10 min, followed by ultrasonic treatment on ice for 10 min and incubation at -20 °C for 1 h. After centrifugation at 12,000 rpm for 15 min at 4 °C, 500 μL of the supernatant was diluted with LC-MS grade water to a final methanol concentration of 60%. The solution was transferred to a fresh Eppendorf tube, filtered through a 0.22 μm membrane, and centrifuged again under the same conditions. Finally, 120 μL of the supernatant was collected into a 2-mL injection vial for metabolomic analysis.

### LC-MS/MS analysis

The metabolomic analysis was conducted using a Waters UPLC I-Class PLUS system coupled with a Xevo G2-XS QTOF high-resolution mass spectrometer (Waters, USA). Chromatographic separation was performed using a Waters Acquity UPLC HSS T3 column (2.1 mm × 100 mm, 1.8 μm). The mobile phases consisted of 0.1% (v/v) formic acid in water (A) and 0.1% (v/v) formic acid in acetonitrile (B) for both positive and negative ionization modes. A 2 μL sample was injected into the system under a gradient elution program (Wang et al., 2016).

Mass spectrometry data were acquired using MassLynx V4.2 (Waters) in MSe mode, allowing simultaneous collection of low-energy and high-energy fragmentation spectra. The collision energy was set to 2V for low-energy and ramped from 10 to 40V for high-energy acquisition, with a scan frequency of 0.2 seconds per spectrum. The ESI ion source parameters were as follows: capillary voltage, 2000V (positive) or -1500V (negative); cone voltage, 30V; ion source temperature, 150°C; desolvation temperature, 500°C; backflush gas flow rate, 50 L/h; and desolvation gas flow rate, 800 L/h.

### Data processing and metabolite identification

Raw LC-MS data were processed using Progenesis QI V2.3 (Nonlinear Dynamics, UK) for peak alignment, retention time correction, baseline filtering, and feature extraction. The main processing parameters included a 5 ppm precursor tolerance, 10 ppm product ion tolerance, and 5% product ion threshold. Features with missing values in more than 50% of the samples were removed, and zero values were replaced with half of the minimum detected intensity. Features with a Progenesis QI identification score below 36 (out of 60) were also excluded (Yang et al., 2021). Metabolite identification was performed by matching MS and MS/MS spectra against the METLIN (Guijas et al., 2018) database and an in-house reference library (Biomarker Biotech, Beijing, China). Identification criteria included precise mass-to-charge ratio (m/z), secondary fragment patterns, and isotope distribution, with molecular ion mass deviation set to <100 ppm and fragment ion deviation set to <50 ppm.

Prior to statistical analysis, data were normalized using Pareto scaling and log-transformed. Differential metabolites were identified using a combination of fold change (FC ≥ 2 or FC ≤ 0.5), t-test significance (*p* < 0.05), and variable importance in projection (VIP > 1) from an orthogonal partial least squares discriminant analysis (OPLS-DA) model (Thévenot et al., 2015). The KEGG database was used for functional annotation of metabolites (Kanehisa and Goto, 2000), while pathway enrichment analysis was conducted using MetaboAnalyst V5.0 (Pang et al., 2021), integrating KEGG pathway mapping. Statistical analyses, including principal component analysis (PCA) and Spearman correlation analysis, were performed to assess metabolomic variation and quality control. To identify key metabolic pathways associated with bacterial wilt, hypergeometric distribution tests were applied to determine the significance of KEGG pathway enrichment.

### Integrated analysis of metabolomic and microbial data

Procrustes analysis was performed to assess the concordance between metabolomic and microbial community data across root-associated niches (McHardy et al., 2013). PCoA was applied separately to the microbial abundance matrix (genus level) using Bayesian distance and the metabolite abundance matrix using Euclidean distance. The first principal coordinates from both datasets were extracted and subjected to Procrustes transformation, minimizing squared deviations to evaluate dataset similarity. O2PLS analysis was used to model intrinsic correlations between microbial and metabolomic datasets, with UV scaling applied before constructing the model (Mayneris-Perxachs et al., 2021). The joint score plot visualized global interactions, while loading values identified key taxa and metabolites, selecting the top 15 based on their contribution to the first two dimensions. Correlation analysis used Spearman’s method to link microbial taxa (phylum level) with dimensionally reduced metabolites, while WGCNA clustered metabolites into co-expression modules to assess microbial-metabolite associations (Kappel et al., 2020). Differential metabolite-microbiome correlations were analyzed without dimensionality reduction to capture direct interactions. Statistical analyses and visualizations were conducted in R using the vegan, OmicsPLS, WGCNA, Hmisc, pheatmap, circlize, igraph, and ggplot2 packages.

## Result

### Spatial dynamics and functional shifts of root microbial communities under bacterial wilt stress

To investigate the spatial dynamics and functional shifts of root-associated microbial communities under bacterial wilt stress, a comprehensive analysis was conducted on microbial diversity and community composition across root-associated niches. The study compared healthy and bacterial wilt-affected potato plants from the same field during a bacterial wilt outbreak. A total of 36 samples were analyzed, including root surrounding, rhizosphere and endosphere niches, using third-generation full-length 16S rRNA and ITS sequencing for epiphytic samples and V3+V4/ITS1 regions for endophytic samples due to primer limitations. Sequencing yielded an average of 13,000 CCS (Circular Consensus Sequencing) reads for 16S rRNA and 12,261 CCS reads for ITS in epiphytic samples, with 400,188 clean reads for 16S rRNA and 87,341 clean reads for ITS in endophytic samples (**Supplementary Tables S1**, **S2**).

**Figure 1:**
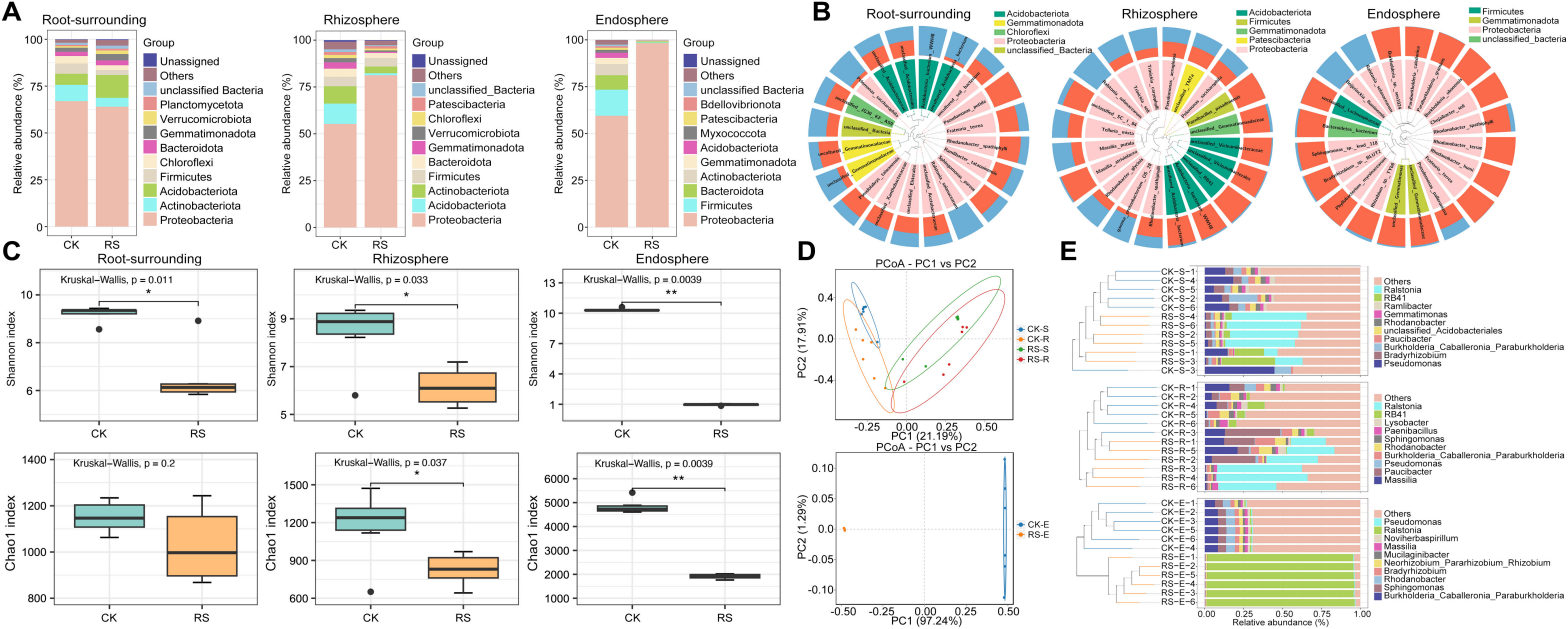
Taxonomic composition, alpha diversity, beta diversity, and hierarchical clustering of bacterial communities across root-associated niches in healthy (CK) and wilt-affected plants (RS). **(A)** Taxonomic composition analysis of bacterial communities at the phylum level, comparing healthy and diseased plant samples across root-surrounding soil, rhizosphere, and endosphere niches. **(B)** Sample community distribution based on phylogenetic trees. The circular tree represents the phylogenetic relationships among taxa, with taxa belonging to the same phylum labeled in the same color. The outer bar plot shows the relative abundance of taxa, with blue bars indicating diseased plant samples and red bars indicating healthy plant samples. **(C)** Alpha diversity analysis (Shannon and Chao1 indices) showing microbial richness and evenness differences between healthy and diseased plants. **(D)** Beta diversity analysis using Bray-Curtis distance-based Principal Coordinate Analysis (PCoA), illustrating the distinct clustering of microbial communities between healthy and diseased groups (S, root surrounding soils; R, rhizosphere; E, endosphere). **(E)** UPGMA clustering tree combined with phylum-level relative abundance bar plots, highlighting phylogenetic relationships and compositional differences between bacterial communities in different root-associated niches. Statistical significance was tested using one-way ANOVA. * and **, significant at P < 0.05 and 0.01, respectively.

Taxonomic analysis revealed distinct microbial compositions across niches. In diseased plants, Proteobacteria dominated the endosphere, comprising over 98% of the microbial community, while healthy plants exhibited greater diversity, with Firmicutes, Bacteroidota, and Actinobacteriota contributing significantly alongside Proteobacteria. In the rhizosphere, diseased samples showed a marked enrichment of Proteobacteria, whereas healthy samples were enriched with Actinobacteriota and Acidobacteriota. In root-surrounding soil, Acidobacteriota were abundant in diseased plants, while Actinobacteriota and Firmicutes was dominant in addition to Proteobacteria in healthy plants (**Figure 1A**). Analyses of the top 20 most abundant bacterial taxa across niches revealed distinct patterns between healthy and wilt-affected plants (**Figure 1B**). In the root-surrounding soil, *R. solanacearum*, *Pelomonas saccharophila*, Acidobacteria bacterium WWH8, and uncultured Acidobacteria bacterium were significantly more abundant in diseased plants, with the latter three taxa being phylogenetically related. In contrast, *Pseudomonas putida* dominated in healthy plants. In the rhizosphere, diseased plants showed increased levels of *R. solanacearum*, along with *Massilia putida*, gamma-proteobacterium OS 28, *Trinickia soli*, *Trinickia caryophylli*, *Rhodanobacter glycinis*, and *Telluria mixta*, all phylogenetically linked to the pathogen Rs. Healthy plants, however, exhibited enrichment of Acidobacteria taxa such as WWH8 and RB41, *Paenibacillus pasadenensis*, and unclassified Vicinamibacteraceae and Gemmatimonadaceae, forming a distinct phylogenetic cluster. In the endosphere, *Beijerinckia fluminensis* and *R. solanacearum* were the only taxa significantly enriched in diseased plants, while all other bacterial taxa were more abundant in healthy plants, highlighting a pronounced microbial shift under bacterial wilt stress.

Alpha and beta diversity analyses revealed significant microbial shifts across root-associated niches under healthy and wilt-affected conditions (**Supplementary Table S3**). Shannon and Chao1 indices showed consistently higher alpha diversity in healthy plants, with differences ranging from non-significant in root-surrounding soil to significant in the rhizosphere and highly significant in the endosphere, indicating that bacterial wilt disrupts bacterial diversity most profoundly closer to the root interior (**Figure 1C**). Beta diversity, analyzed using Bray-Curtis distance matrices in principal coordinate analysis (PCoA), revealed clear separation between healthy and diseased groups. In epiphytic samples, the first coordinate distinguished health status, while the second separated rhizosphere from root-surrounding soil (PERMANOVA: *R* = 0.5177, *p* = 0.001; ANOSIM: *R²* = 0.3244, *p* = 0.001). In endosphere samples, healthy and diseased groups were distinctly separated (PERMANOVA: *R* = 1, *p* = 0.001; ANOSIM: *R²* = 0.9724, *p* = 0.001), reflecting significant bacterial differences (**Figure 1D**). UPGMA clustering and genus-level abundance plots confirmed these patterns, showing pathogenic *Ralstonia* dominating the diseased group, increasing from rhizosphere to endosphere, where it became nearly exclusive. Genera such as *Pseudomonas*, *Massilia*, and *Burkholderia*, which dominated healthy niches, sharply declined in diseased samples, becoming nearly absent in the endosphere (**Figure 1E**). These findings demonstrate that bacterial wilt profoundly alters bacterial diversity and composition, with the most severe disruptions observed in the endosphere, where beneficial taxa are replaced by pathogen dominance.

Functional predictions using FAPROTAX further highlighted distinct ecological roles of bacterial communities (**Supplementary Figure S1**). In root-surrounding soil and rhizosphere, diseased plants showed functional enrichment in plant pathogenesis and organic matter degradation (e.g., chitinolysis, ureolysis), and fermentation, indicating pathogen-driven decomposition. Conversely, healthy plants exhibited functions linked to chemoheterotrophy, aerobic chemoheterotrophy, nitrate reduction, and nitrogen fixation, underscoring their contribution to nutrient cycling. Within the endosphere, healthy plants maintained functionally diverse microbial communities involved in nitrogen fixation, fermentation, and parasitic or symbiotic interactions, while diseased plants were dominated by pathogen-associated functions. These findings illustrate a functional shift from beneficial microbial processes in healthy plants to pathogen-centric activities in diseased plants, reflecting the impact of bacterial wilt on root-associated microbial ecology.

In terms of fungal compositions, while root-surrounding fungal composition exhibited minor variations, the rhizosphere displayed significant divergence, with *Gymnoascus reessii* and *Fusarium foetens* markedly enriched in diseased plants, whereas *Penicillium cremeogriseum*, dominant in healthy plants, showed a sharp decline. Endophytic fungal composition remained largely stable, except for a significant increase in *Fusarium circinatum* abundance in the diseased plants. These findings suggest that *R. solanacearum* infection promotes the enrichment of *Fusarium* species in both the rhizosphere and endosphere (**Supplementary Figure S2**). Alpha diversity analysis of fungal communities revealed contrasting patterns across root-associated niches (**Supplementary Figure S3**). Both root-surrounding and rhizosphere fungal diversity were higher in healthy plants compared to diseased plants, whereas in the endosphere, Shannon and Chao1 indices were elevated in diseased plants. This suggests that bacterial wilt infection suppresses fungal diversity in external root compartments while promoting a more diverse fungal community within the endosphere. Additionally, the clear separation of fungal communities in PCoA between healthy and diseased groups across all root-associated niches indicates that bacterial wilt infection induces a distinct restructuring of the fungal microbiome, leading to significant compositional shifts both in root-external and endophytic compartments (**Supplementary Figure S4**).

Based on phenotype prediction using FUNGuild, a tool that classifies fungal taxa into ecologically meaningful guilds, significant shifts in fungal functional guilds were observed across all root-associated compartments in response to *R. solanacearum* infection (**Supplementary Figure S5**). In the root-surrounding soil, disease-associated enrichment was observed in endophytes, arbuscular mycorrhizal fungi, and several saprotrophic and animal pathogenic guilds. Conversely, wood and leaf saprotrophs significantly declined, suggesting that typical decomposers were suppressed under pathogen pressure. In the rhizosphere, the diseased plants showed a marked increase in saprotrophs and related guilds. However, beneficial guilds such as arbuscular mycorrhizal fungi, ectomycorrhizal fungi, and wood saprotrophs were significantly reduced. Endophytic fungal communities exhibited a notable shift from mutualistic to saprotrophic and pathogenic guilds under disease stress. In the diseased plants, significant enrichment was observed in saprotrophs, plant and animal pathogens, and epiphytic fungi. In contrast, the healthy plants harbored higher abundances of mycorrhizal guilds (e.g., Ericoid Mycorrhizal, Arbuscular Mycorrhizal) and endophytic-lichen symbionts, indicating a more stable and beneficial symbiotic structure. These findings indicate that pathogen stress drives a functional reorganization of root-associated fungal communities, shifting from mutualistic and decomposer guilds to an increased prevalence of opportunistic saprotrophs and potential pathogens.

### Network topology and core microbial taxa driving community stability

**Figure 2:**
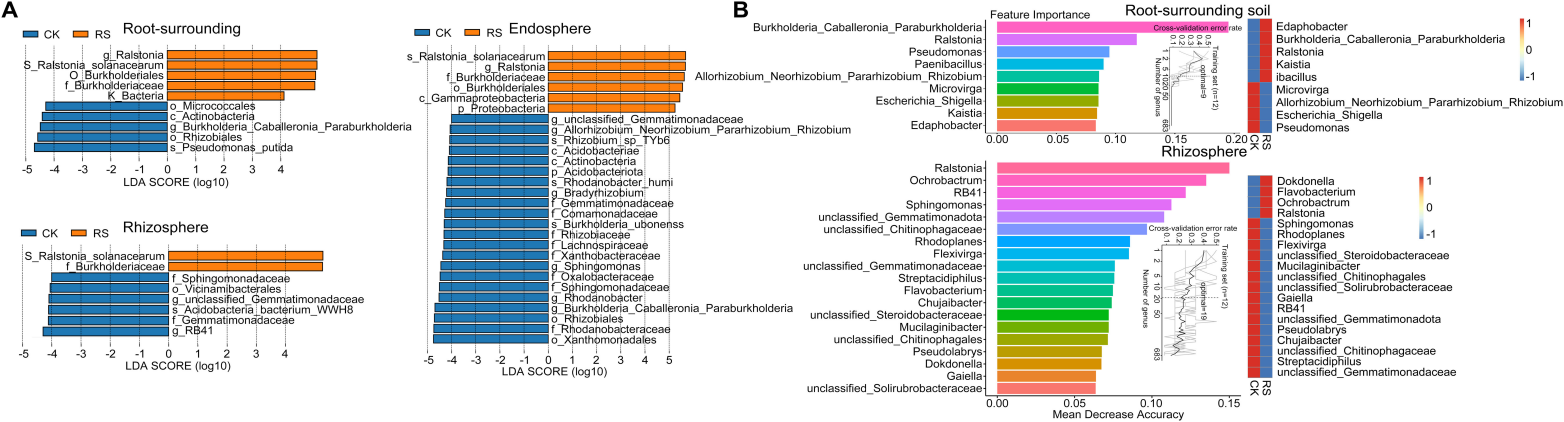
Differential microbial taxa identified by LEfSe and Random Forest analysis. **(A)** LEfSe (Linear Discriminant Analysis Effect Size) analysis showing differentially abundant bacterial taxa between healthy (CK) and diseased plants (RS) across root-associated niches. The LDA score (log10) represents the effect size of each biomarker taxon, with positive values indicating taxa enriched in diseased plants and negative values indicating taxa enriched in healthy plants. Only taxa with an LDA score > 4 and *p* < 0.05 are shown. **(B)** Random Forest model detecting key bacterial taxa that serve as biomarkers distinguishing microbial communities in healthy and diseased plants. The top 9 bacterial genera in root-surrounding soil (top panel) and the top 19 genera in the rhizosphere (bottom panel) were identified based on their importance in classification accuracy. Biomarker taxa are ranked in descending order of importance to the accuracy of the model. The inset represents the 10-fold cross-validation error as a function of the number of input taxa used to differentiate healthy and diseased plants in order of variable importance. The heatmap displays the relative abundances of the selected predictive biomarker genera across the two plant health conditions.

LEfSe analysis revealed distinct biomarker taxa across niches between healthy and bacterial wilt-affected plants (**Figure 2A**). In diseased plants, biomarkers were limited to the pathogen *R. solanacearum*, its genus *Ralstonia*, and its family Burkholderiaceae across all three niches. In contrast, healthy plants exhibited a broader range of enriched taxa. In the rhizosphere, biomarkers included Sphingomonadaceae, Vicinamibacterales, Gemmatimonadaceae, Pyrinomonadaceae, and *RB41*. The root-surrounding soil was enriched with taxa such as Micrococcales, Rhizabiales, *Burkholderia_Caballeronia_Paraburkholderia*, and *Pseudomonas putida*. The healthy endosphere harbored a diverse set of biomarkers, including species like *Burkholderia ubonensis*, *Rhodanobacter humi*, and *Rhizobium* sp. *TYb6*, highlighting the healthy group’s microbial diversity and functional enrichment.

For the epiphytic samples, Random Forest analysis further identified key bacterial taxa differentiating healthy and diseased groups (**Figure 2B**). The analysis was conducted across multiple taxonomic levels, with a 10-fold cross-validation used to determine the optimal classification model. At the genus level, this approach achieved the highest classification accuracy with 9 genera in root-surrounding soil and 19 in the rhizosphere. The most critical biomarkers for the root-surrounding soil included three genera from the family Burkholderiaceae (*Burkholderia*_*Caballeronia*_*Paraburkholderia*). Genera such as *Pseudomonas*, *Paenibacillus*, and *Rhizobium*, alongside pathogenic *Ralstonia*, were highlighted as key indicators, aligning with LEfSe results. In the rhizosphere, *Ralstonia* emerged as the most significant biomarker for diseased plants, while *RB41*, *Sphingomonas*, and unclassified Gemmatimonadota, enriched in healthy samples, were also confirmed. Random Forest additionally identified genera such as *Ochrobactrum*, unclassified Chitinophagaceae, *Rhodoplanes*, *Flavobacterium*, and *Chujaibacter*, which were not detected in LEfSe analysis. Together, these results demonstrate bacterial wilt’s significant impact on root-associated microbial communities, with genus-level biomarkers serving as robust indicators of microbial shifts in external root niches.

**Figure 3:**
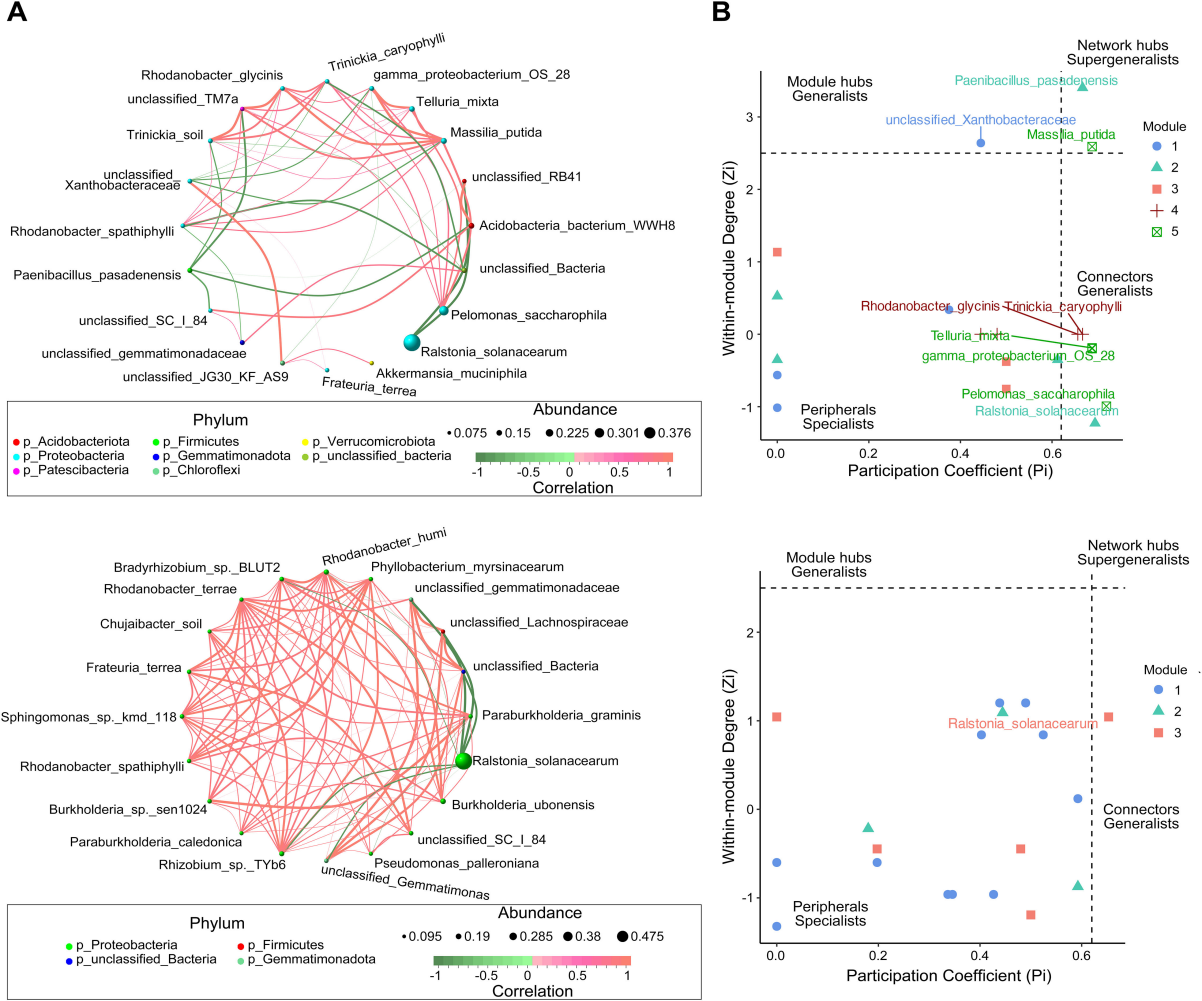
Co-occurrence network analysis of microbial communities in the epiphytic (top) and endosphere (bottom) niches of healthy and diseased plants. **(A)** Microbial co-occurrence networks, where each node represents a species, and the node size corresponds to the mean abundance of the species. Edges represent significant correlations between species, with edge thickness indicating the strength of correlation. Red edges denote positive correlations, whereas green edges indicate negative correlations. **(B)** Zi-Pi plot illustrating the topological roles of operational taxonomic units (OTUs) within the network. Each symbol represents an OTU, with classification thresholds of *Zi* > 2.5 and *Pi* > 0.62 (Olesen et al., 2007). Species identified as Connectors, Module hubs, and Network hubs are labeled.

The analysis of bacterial co-occurrence networks across root-associated niches revealed distinct structural and functional dynamics (**Figure 3A**, **Supplementary Table S4**). The endosphere network exhibited the highest complexity and stability, with the most nodes and edges, the highest average degree (10.0), and strong local connectivity (the shortest average path length (1.18) and the highest clustering coefficient 0.88), enabling efficient information transfer and cooperative subcommunity formation. Its low modularity (0.15) suggested a uniform structure focused on overall stability. In contrast, the rhizosphere network displayed the highest modularity (0.43), indicating functional differentiation with tightly connected submodules that enhanced adaptability under stress. It also maintained strong connectivity (relatively short average path length (1.49) and high clustering coefficient (0.66)) and efficient information flow, while its low betweenness centralization (0.02) highlighted a balanced influence among nodes. The root-surrounding soil network was more dispersed, with the longest average path length (1.86) and the lowest modularity (0.09), reflecting reduced connectivity efficiency. However, its reliance on a few critical nodes, indicated by higher degree (0.32) and betweenness centralization (0.20), suggested vulnerability to disruptions.

Zi-Pi analysis of the epiphytic and endosphere networks further revealed differences in regulatory dynamics (**Figure 3B**). The epiphytic network featured higher complexity and diverse key nodes, with pathogen *R. solanacearum* acting as a connector counterbalanced by beneficial taxa such as Acidobacteria WWH8 and RB41. Notable hubs, including *Massilia putida* and *Paenibacillus pasadenensis*, played essential roles in regulating network dynamics. *M.putida* exhibited positive correlations with eight nodes, including gamma-proteobacterium OS 28 and *Telluria mixta*, and negative interactions with unclassified Xanthobacteraceae. Similarly, *Pelomonas saccharophila*, as a key inter-module connector, positively correlated with six nodes, emphasizing its regulatory significance. In contrast, the endosphere network was dominated by *R. solanacearum*, which served as the sole connector with negative effects on beneficial taxa such as *Rhizobium* sp. TYb6, *Rhodanobacter humi* and *Burkholderia ubonensis*. Despite this pathogen-centric control, positive interactions among the remaining 19 nodes maintained localized cohesion. These findings highlight the epiphytic network’s reliance on diverse nodes for regulatory balance, whereas the endosphere network demonstrates centralized, pathogen-driven dynamics under bacterial wilt stress.

### Pathogen-induced metabolic reprogramming in root-associated niches

To explore metabolic differences between healthy and bacterial wilt-affected plants across root-associated niches, a metabolomic analysis of 36 samples was performed using an LC-QTOF platform, detecting 8,766 peaks and annotating 1,596 metabolites. Principal component analysis (PCA) of normalized data (unit variance scaling) revealed distinct metabolic profiles, with significant separation observed in the endosphere, indicating marked pathogen-induced metabolic shifts, while differences in the root-surrounding soil and rhizosphere were less distinct, highlighting the localized impact within root tissues (**Supplementary Figure S6**). Correlation analysis and heatmap clustering confirmed niche-specific metabolic variations, with endosphere metabolites exhibiting distinct functional profiles compared to external root niches (**Supplementary Figure S6**). KEGG pathway annotation identified significant enrichment in secondary metabolite biosynthesis (e.g., glucosinolates, alkaloids including tropane, indole and isoquinoline, phenylpropanoids, and terpenoids) as well as pathways related to carbohydrate and lipid metabolism, providing insights into the reconfiguration of plant and microbial metabolic networks under pathogen stress (**Supplementary Figure S7**).

Comparative analysis of metabolic profiles between healthy and diseased plants revealed significant shifts in metabolite abundance across root-associated niches (**Supplementary Figure S8**). In the rhizosphere, 512 metabolites were upregulated and 86 downregulated in diseased plants. Upregulated metabolites, such as kynurenic acid, phosphatidylcholine (PC(14:1(9Z)/16:1(9Z))), and 5,7-dimethoxyflavone, were linked to defense responses, secondary metabolism, and antioxidative stress, highlighting active responses to pathogen invasion. Conversely, downregulated metabolites, including glyceric acid and 3-carboxy-2-hydroxyadipic semialdehyde, reflected suppressed carbon metabolism, while reductions in tropane and flavonoids (e.g., butterfly flavone A) indicated weakened antimicrobial pathways. In the endosphere, bacterial wilt induced significant metabolic reprogramming, with 299 metabolites upregulated and 483 downregulated. Upregulated metabolites, such as hydrangeifolin I, isoliquiritin apioside, arachidonic acid, and diacylglycerol, suggested enhanced disease resistance signaling, antioxidative activity, membrane remodeling, and signal transduction. Downregulated metabolites, including camalexin, thiamine derivatives (e.g., 2-(alpha-hydroxypropyl)thiamine diphosphate), and tryptophan-related products, indicated suppressed primary metabolism and antimicrobial pathways. The reduction of antibiotic-related metabolites, such as 2,3-dihydrothiazoloquinone, further highlighted weakened pathogen inhibition. Comparisons between rhizosphere and endosphere in diseased plants provided additional insights into pathogen-driven reprogramming. The endosphere showed 748 upregulated and 352 downregulated metabolites compared to the rhizosphere, with elevated levels of antimicrobial metabolites such as verruculogen, paclitaxel, and aurachin A, reflecting intensified chemical defenses. Increased glutamylcysteine and peptide metabolites indicated enhanced signaling and antioxidative responses, while reduced quinolinic acid and 11a-hydroxytetracycline suggested suppressed nucleic acid metabolism and antibiotic synthesis due to pathogen competition and resource reallocation.

**Figure 4:**
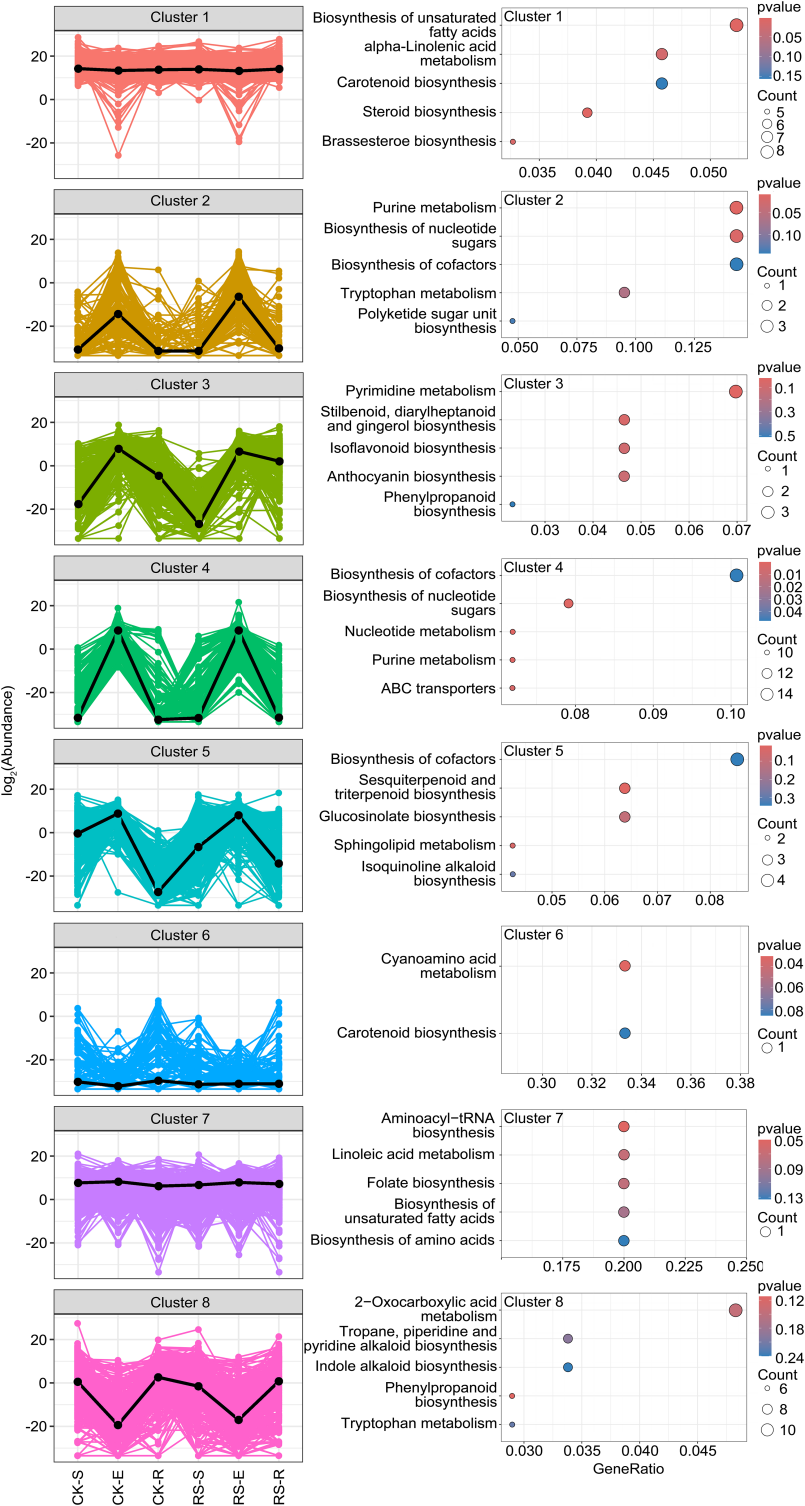
K-means clustering of differential metabolites and KEGG functional enrichment analysis. The left panel showed K-means clustering of differential metabolites identified eight distinct clusters. The clustering pattern reveals niche- and infection-specific metabolic shifts in root-associated compartments. The right panel presented KEGG functional enrichment analysis of metabolites in each cluster. The top five enriched KEGG pathways are shown for each cluster. Pathway significance was determined based on enrichment scores.

K-means clustering of differential metabolites identified eight distinct clusters, each reflecting niche- and infection-specific metabolic reprogramming across root-associated compartments (**Figure 4**). Clusters 1 and 7 represented core metabolic pathways with high metabolite abundance across all samples, regardless of infection status. These metabolites were enriched in pathways such as biosynthesis of unsaturated fatty acids, alpha-linolenic acid metabolism, and brassinosteroid biosynthesis, essential for maintaining cellular integrity, membrane stability, and basal metabolic processes. Cluster 3 and Cluster 5 showed similar abundance patterns that were associated with pathogen-induced responses, particularly in the rhizosphere and endosphere. Cluster 3 metabolites, highly enriched in the infected endosphere and rhizosphere, were linked to pyrimidine metabolism, phenylpropanoid biosynthesis, and gingerol-related pathways, showing their roles in defense and stress signaling. Cluster 5 metabolites, predominantly enriched in the infected endosphere and elevated in the infected rhizosphere compared to the healthy rhizosphere, were associated with sesquiterpenoid and triterpenoid biosynthesis, glucosinolate biosynthesis and sphingolipid metabolism, critical for antimicrobial activity and cell wall reinforcement. Clusters 2, 4, and 8 exhibited niche-specific patterns. Cluster 2 metabolites, abundant in both healthy and infected endosphere, were enriched in purine metabolism, nucleotide sugar biosynthesis, and tryptophan metabolism, suggesting their role in core metabolic processes essential for endosphere stability. Cluster 4 metabolites, consistently abundant in the endosphere, were involved in nucleotide sugar biosynthesis and ABC transporters, highlighting their role in maintaining cellular homeostasis and metabolic regulation. In contrast, Cluster 8 metabolites were enriched in root-surrounding soil and rhizosphere but depleted in the endosphere, reflecting their roles in 2-oxocarboxylic acid metabolism, tropane, piperidine and pyridine alkaloid biosynthesis, indole alkaloid and phenylpropanoid biosynthesis, indicating their role in antimicrobial activity, oxidative stress response, and sustaining external microbial communities. These findings highlight distinct metabolic shifts under bacterial wilt stress: Clusters 3 and 5 emphasize the dynamic antimicrobial and signaling responses in the rhizosphere and endosphere, Clusters 2 and 4 focus on endosphere stability, and Cluster 8 showed a crucial role in stabilizing root-associated microbial interactions and environmental adaptation, fostering a balanced rhizosphere community by supporting beneficial microbes and suppressing pathogens. This coordinated metabolic reprogramming provides insights into the biochemical foundations of plant-microbe-pathogen interactions.

### Integrated insights into microbiome-metabolome interactions shaping plant health

**Figure 5:**
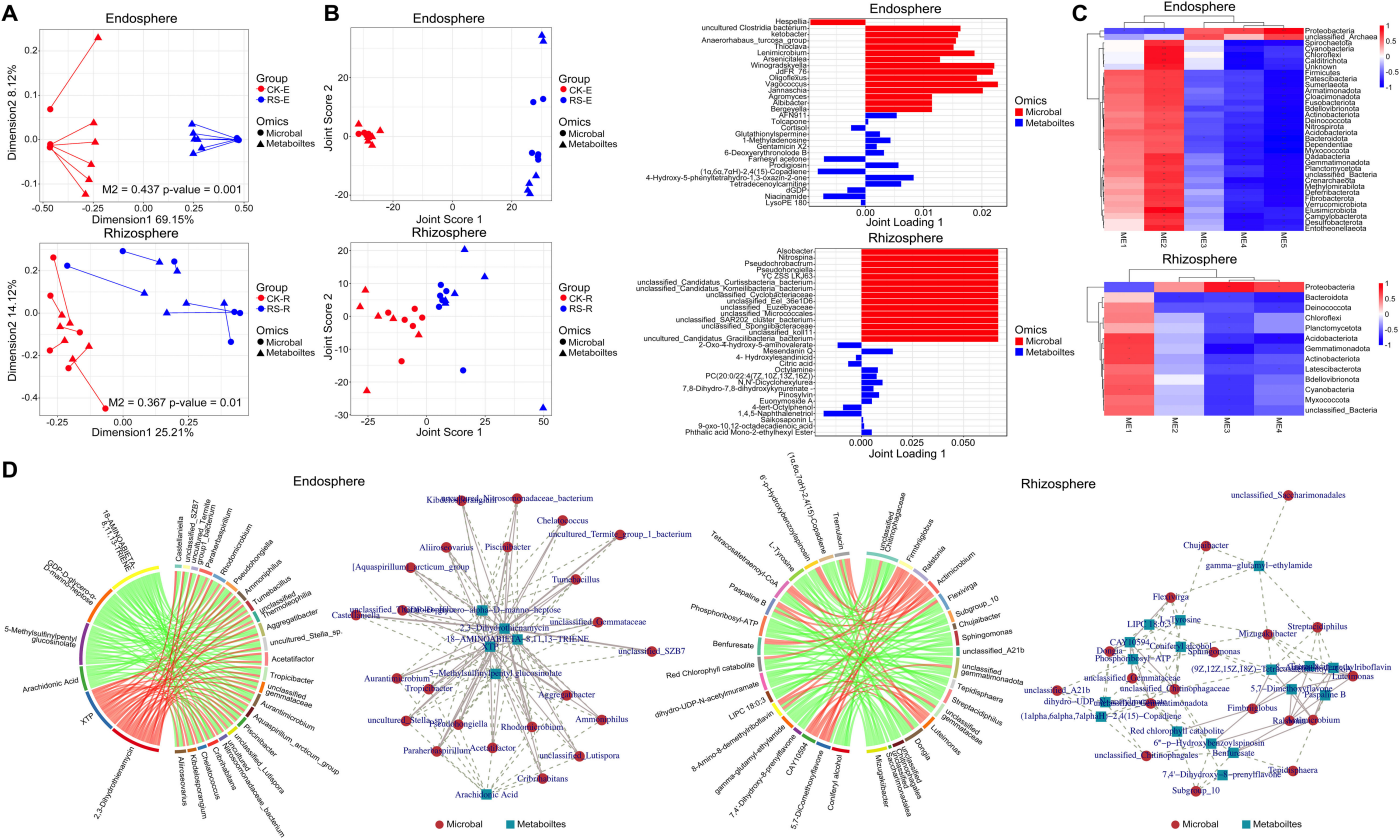
Integrated analysis of microbiome-metabolome interactions across root-associated niches. **(A)** Procrustes analysis showing the alignment between microbial community composition and metabolomic profiles in healthy and diseased plants. The upper section represents the endosphere, the lower section represents the rhizosphere. Longer arrow lengths indicated a dispersed and complex microbiome-metabolome relationship, whereas shorter and aligned arrows suggested convergence of microbial and metabolic networks. **(B)** O2PLS analysis of microbial and metabolite associations. The left panel displays the joint score plot, showing sample distribution based on O2PLS modeling of microbial and metabolic data. The right panel presents loading plots, selecting the top 15 metabolites and microbial taxa with the highest loadings in the first two dimensions, highlighting key drivers of infection-induced metabolic shifts. The upper section corresponds to the endosphere, and the lower section to the rhizosphere. **(C)** WGCNA-based correlation analysis heatmap, illustrating module-trait relationships between microbial taxa and metabolite modules in the endosphere (upper) and rhizosphere (lower). **(D)** Correlation analysis between differential metabolites and microbial genera in the endosphere and rhizosphere. The left panel displays a chord diagram ranking the top 30 microbe-metabolite correlations by correlation coefficient. The right panel presents a co-occurrence network, where nodes represent metabolites or microbial genera, and edges indicate significant correlations, with line thickness reflecting correlation strength.

Procrustes analysis highlighted distinct microbiome-metabolome interactions across root-associated niches under healthy and diseased conditions (**Figure 5A**). In the endosphere, healthy plants exhibited more diverse and dispersed arrow directions with longer lengths, reflecting a complex and varied relationship between microbial communities and metabolic functions. Conversely, diseased plants showed concentrated arrow directions and shorter lengths, indicating pathogen-induced reshaping of metabolic networks and microbial composition to adapt to the endosphere environment. In the rhizosphere, healthy plants displayed relatively concentrated arrows, suggesting stable microbiome-metabolome interactions, whereas diseased plants had more dispersed arrows, reflecting dynamic reorganization in response to pathogen stress. These results demonstrate the pathogen’s niche-specific strategies for metabolic and microbial adaptation.

O2PLS analysis further elucidated cross-omic relationships, revealing stable microbiome-metabolome coordination in healthy states and significant remodeling under pathogen stress (**Figure 5B**). In the endosphere, healthy plants showed compact clustering in joint score plot, indicating coordinated microbial and metabolic stability, while diseased plants exhibited dispersed distributions, reflecting disrupted microbial-metabolic networks following pathogen invasion. Key associations included dominant taxa *Hespellia* with Niacinamide and LysoPE 180 in healthy plants, supporting antioxidative and metabolic functions, and Uncultured_Clostridia_bacterium and *Ketobacter* with Glutathionylspermine and Gentamicin X2 in diseased plants, indicating enhanced antimicrobial and antioxidative responses. In the rhizosphere, a critical interface for pathogen-host interactions, both healthy and diseased plants displayed relatively dispersed distributions in joint score plot, underscoring the highly dynamic interplay between microbiomes and metabolomes. Healthy plants enriched metabolites like Citric acid and 4-Hydroxybutanoic acid, reflecting stable carbon metabolism and energy balance indicative of a stable environment, while infected plants exhibited chemical defense-related metabolites such as Mesendanin Q and Saikosaponin L. Pathogen invasion also enriched taxa like *Pseudochrobactrum*, *Alsobacter*, and *Nitrospina*, suggesting a role in infection-driven metabolic remodeling by interacting with defense-related metabolites. These findings underscore distinct pathogen-induced changes in microbiome-metabolome coordination across niches.

WGCNA-based correlation analysis revealed distinct metabolite-microbe interaction patterns in the endosphere and rhizosphere, reflecting niche-specific responses to bacterial wilt infection (**Figure 5C**). In the endosphere, the ME2 module, enriched in energy metabolism and amino acid metabolism-related compounds, including Citric acid and L-Glutamine, showed a negative correlation with Proteobacteria, but a significant positive correlation with multiple phyla such as Firmicutes, Acidobacteriota, and Actinobacteriota (*r* > 0.4, *p* < 0.05). Module ME5 was enriched in Gentamicin X2, Glutathionylspermine and Tropane alkaloids, a class of secondary metabolites closely associated with plant defense mechanisms against microbial pathogens. This module was positively correlated with Proteobacteria (*r* > 0.5, *p* < 0.05) but negatively correlated with other bacterial phyla, suggesting that pathogen-induced stress may promote the accumulation of these antimicrobial compounds. Module ME4 was linked to the neomycin biosynthesis pathway, which is potentially involved in synergistic antimicrobial activity. Similar to ME5, this module displayed a strong positive correlation with Proteobacteria (*r* > 0.5, *p* < 0.05) while showing a negative correlation with other microbial groups, reinforcing the role of these metabolites in pathogen defense. In the rhizosphere, the ME3 module exhibited a strong positive correlation with Proteobacteria (*r* = 0.8, *p* < 0.01) and a negative correlation with Gemmatimonadota (*r* = -0.65, *p* < 0.05), emphasizing the role of Proteobacteria in carbon and nitrogen cycling via metabolites like Citric acid and Saikosaponin L. Bacteroidota, which negatively correlated with multiple modules, showed reduced metabolic interactions, indicating limited involvement in pathogen-driven metabolic shifts. The ME1 module positively correlated with Actinobacteriota (*r* = 0.72, *p* < 0.05) and was enriched in metabolites such as Prostaglandin E2 and Mesendanin Q, associated with oxidative stress and plant defense responses. These findings revealed distinct metabolic reprogramming under pathogen stress: the endosphere prioritizes antimicrobial metabolite upregulation while suppressing energy and nitrogen metabolism, whereas the rhizosphere undergoes metabolic shifts closely aligned with microbial community dynamics, with taxa such as Proteobacteria and Actinobacteriota shaping ecosystem functionality.

Correlation analysis of differential metabolites and microbial genera in the endosphere and rhizosphere revealed distinct differences in metabolite-microbe interactions between healthy and diseased plants under bacterial wilt stress (**Figure 5D**). In the endosphere, antimicrobial metabolites such as 5-Methylsulfinylpentyl glucosinolate and Arachidonic acid showed strong negative correlations with *Aggregatibacter* and *Tropicibacter* (*r* < -0.7, *p* < 0.01), suggesting their role in microbial suppression and pathogen defense. Arachidonic acid, an inflammation-associated lipid, may also mediate host immune responses, influencing microbial dynamics. Conversely, XTP and 2,3-Dihydrothienamycin exhibited strong positive correlations with *Rhodomicrobium*, *Acetatifactor*, and *Aliiroseovarius* (*r* > 0.7, *p* < 0.01), indicating enhanced microbial metabolic activity and potential antimicrobial compound reorganization under infection. Network analysis positioned XTP as a core node with dense microbial associations, reflecting its central role in infection-induced metabolic shifts, while antimicrobial metabolites such as Arachidonic acid and 5-Methylsulfinylpentyl glucosinolate were located at peripheral nodes, exerting targeted inhibitory effects on specific microbes. In the rhizosphere, Paspaline B and 8-Amino-8-demethylriboflavin correlated positively with *Ralstonia* and its associated genera, *Luteimonas* and *Actimicrobium*, suggesting their involvement in pathogen proliferation, either as nutrient sources or regulatory signals. Conversely, L-Tyrosine and Coniferyl alcohol displayed strong negative correlations with genera such as *Dongia* and *Sphingomonas*, implying their role in suppressing pathogen activity and maintaining ecosystem stability. Network analysis identified Benfuresate and Red chlorophyll catabolite as central metabolites with extensive microbial associations, playing dual roles in rhizosphere regulation by promoting microbial community function while inhibiting potential pathogens. In contrast, L-Tyrosine and Coniferyl alcohol were positioned at the network periphery, with fewer but strong targeted associations, indicating their localized regulatory significance. These findings suggest that antimicrobial metabolites function through negative associations to inhibit pathogens, while metabolically active compounds such as XTP and 2,3-Dihydrothienamycin participate in microbial metabolic restructuring. In the rhizosphere, core metabolites act as ecological regulators balancing microbial activity and pathogen suppression, while peripheral metabolites mediate targeted interactions, highlighting the complex metabolic-microbial interplay shaping plant-associated ecosystems.

## Discussion

Bacterial wilt imposes profound disruptions on root-associated microbial communities, leading to extensive metabolic and ecological reprogramming. The observed niche-specific shifts in microbial diversity and composition suggest that the pathogen-driven restructuring is not a uniform process but rather a spatially regulated adaptation that varies across root-associated compartments. The endosphere exhibited the most severe microbial collapse, with *R. solanacearum* dominating the microbial niche, while other non-dominant commensal taxa were largely reduced. This suggests that pathogen invasion selectively eliminates or suppresses competitive microbiota, potentially through allelopathic interactions or immune suppression strategies that allow its unchecked proliferation. Similar microbial dominance by *R. solanacearum* has been reported in various plant systems, where it outcompetes native microbiota through quorum sensing, effector-mediated suppression, and secretion of antimicrobial compounds (Vannier et al., 2019). The replacement of a functionally diverse microbiome with a single pathogen further implies that the endosphere is a particularly vulnerable niche under pathogen stress, as it lacks the buffering effects of external microbial interactions found in the rhizosphere. The rhizosphere, in contrast, displayed a more complex microbial response, with increased modularity and functional differentiation, suggesting that microbial networks undergo significant restructuring under pathogen invasion. While Proteobacteria exhibited significant enrichment in diseased plants, taxa such as Acidobacteriota and Actinobacteriota, which were dominant in healthy plants, showed reduced interactions, likely due to shifts in root exudation profiles and altered nutrient dynamics. Previous studies have highlighted that plants can actively modulate rhizosphere microbiomes through exudate-driven selection, promoting beneficial taxa while suppressing pathogenic consortia (Chaparro et al., 2014). The depletion of Acidobacteriota, a group commonly associated with carbon and nitrogen cycling, suggests that pathogen-induced metabolic shifts alter rhizosphere nutrient availability, potentially reducing microbial functional redundancy and thereby facilitating pathogen colonization (Gonçalves et al., 2024).

Metabolomic analysis revealed substantial pathogen-induced metabolic reprogramming, particularly in the endosphere, where upregulation of antimicrobial and oxidative stress-related metabolites occurred alongside suppression of primary metabolism. The enrichment of arachidonic acid, an inflammation-associated lipid, and Gentamicin X2, an antimicrobial compound, suggests that the plant actively engages in chemical defense mechanisms against pathogen stress (Dedyukhina et al., 2014). However, the concomitant increase in Gflutathionylspermine, a redox-balancing metabolite, and its correlation with Ketobacter in diseased plants indicate that oxidative stress plays a central role in disease progression. This aligns with previous findings that oxidative bursts triggered by pathogen invasion lead to metabolic shifts that either enhance immunity or drive tissue degradation (Torres et al., 2006; Lehmann et al., 2015). The depletion of camalexin, a key phytoalexin, in diseased plants suggests that the pathogen may actively suppress host antimicrobial responses, either through effector-mediated immune modulation or by redirecting metabolic pathways to favor its proliferation, a mechanism observed in bacterial wilt infections in ginger and tobacco (Dang et al., 2023; Li et al., 2017). In the rhizosphere, metabolic shifts were tightly linked to microbial composition, with Proteobacteria forming dominant associations with metabolites involved in carbon and nitrogen cycling, such as citric acid and saikosaponin L. This suggests that pathogen infection alters root exudation patterns, potentially benefiting fast-growing copiotrophic microbes while suppressing slow-growing oligotrophs. The enrichment of antibiotic-related metabolites such as verruculogen and aurachin A in the infected endosphere further suggests an adaptive response aimed at microbial competition and pathogen inhibition (Kruth and Nett, 2023). Interestingly, the downregulation of nucleic acid metabolism-related metabolites, such as quinolinic acid, may indicate a shift in microbial community dynamics, where pathogen-driven suppression of specific biosynthetic pathways limits microbial proliferation and facilitates its own dominance. Similar metabolic trade-offs have been observed in other soilborne plant-pathogen interactions, where pathogen-induced metabolic shifts alter rhizosphere nutrient fluxes, thereby favoring disease-promoting microbiomes (Berendsen et al., 2012; Mendes et al., 2018). For example, in ginger and tobacco, infection led to reduced alpha diversity, overrepresentation of Proteobacteria, and enrichment of secondary metabolites involved in defense and oxidative stress responses (Dang et al., 2023; Li et al., 2017). However, distinct metabolites such as saikosaponin L and XTP identified in our potato system highlight host-specific metabolic responses. These comparative insights suggest that while *R. solanacearum* employs conserved infection strategies, host metabolic plasticity and genotype contribute significantly to the disease outcome and microbiome restructuring.

To better understand how such metabolic responses may influence microbial dynamics, we conducted network-level analyses to explore potential key mediators in these interactions. Network analysis highlighted key metabolites that acted as central regulators of microbial interactions. XTP, a nucleotide metabolism intermediate, displayed extensive positive associations with multiple microbial genera, suggesting that pathogen invasion enhances microbial metabolic activity, likely as a response to environmental stress. This observation aligns with studies showing that nucleotide metabolism is critical in microbial adaptation to pathogen-induced stress and energy reallocation (Fitzsimmons et al., 2018). Conversely, antimicrobial metabolites such as 5-Methylsulfinylpentyl glucosinolate and arachidonic acid exhibited strong negative correlations with specific genera, highlighting their selective suppression of pathogenic taxa. The strategic positioning of these metabolites at the network periphery suggests that antimicrobial activity is not broadly distributed but rather targeted, reinforcing the concept that plants employ selective chemical defenses against microbial invaders (Shastri and Kumar, 2019). While our integrated analyses revealed strong correlations between specific microbial taxa and metabolite abundance, the exact source of these metabolites remains ambiguous. Compounds like camalexin and arachidonic acid are typically plant-derived and reflect host immune activation, whereas others such as Gentamicin X2 may be produced by microbial symbionts. The co-occurrence patterns suggest functional associations but do not establish causality. Future studies employing isotope labeling or metatranscriptomics are needed to disentangle plant versus microbial contributions to the metabolite pool and clarify the causal direction of these interactions.

The microbiome-metabolome co-analysis provided insights into the broader ecological implications of these shifts. Procrustes and O2PLS analyses revealed that microbial and metabolic networks in healthy plants were more diverse and spatially dispersed, whereas diseased plants exhibited a more tightly coordinated structure, likely driven by pathogen-imposed constraints. The strong correlation between antimicrobial metabolites and specific bacterial taxa in infected plants suggests that metabolic reprogramming is not merely a passive response but an active, pathogen-mediated process that restructures microbial ecosystems. This is consistent with findings in other plant-pathogen interactions, where the metabolic landscape is reshaped to either support pathogen proliferation or elicit defense responses (Sasse et al., 2018; Lyu et al., 2019). These microbiome–metabolome associations are likely governed by complex molecular mechanisms. For instance, *R. solanacearum* delivers a suite of type III secretion system (T3SS) effectors that modulate host immune responses and interfere with hormone signaling, such as salicylic acid (SA) and jasmonic acid (JA) pathways (Nakano and Mukaihara, 2019). This immunosuppression can alter root exudate composition and local nutrient availability, thereby reshaping the chemical environment and selecting for specific microbial assemblages taxa with specific metabolic capabilities, including the utilization of defense-related secondary metabolites such as phenolics, flavonoids, and saponins (Yang et al., 2023). These changes may favor the enrichment of antimicrobial or copiotrophic bacteria. Similarly, *R. solanacearum* infection was shown to alter tobacco root exudate composition, notably increasing the secretion of caffeic acid, which selectively suppressed pathogen growth and enriched antagonistic rhizosphere bacteria (Li et al., 2021). These mechanisms emphasize the active and dynamic interplay between host defense, pathogen virulence, and microbial community restructuring.

The selective enrichment of potentially beneficial taxa such as *Sphingomonas*, *Paenibacillus*, and *Burkholderia* in healthy plants suggests that microbiome engineering approaches, such as targeted probiotic applications or root exudate manipulation, could enhance disease resistance (Compant et al., 2019; Wei et al., 2020). These strategies, however, must account for environmental variables such as soil physicochemistry and local microbial ecology to be field-applicable. Additionally, the identification of microbial-regulating metabolites such as XTP and saikosaponin L opens opportunities for metabolic priming—either through foliar sprays or soil amendments—to enhance host immunity. Future studies should focus on elucidating the molecular mechanisms underlying these interactions, particularly how pathogen-induced metabolic shifts shape microbial networks at a functional level. By integrating multi-omic approaches, it may be possible to develop predictive models for microbiome resilience and pathogen suppression. Meanwhile, large-scale validation in field trials is essential to assess persistence, colonization efficiency, and crop yield impact under natural pathogen pressures.

## Conclusion and recommendation

This study provides a comprehensive overview of the spatial dynamics of microbial and metabolic responses in potato root-associated niches under bacterial wilt stress. The results demonstrate that *R. solanacearum* infection induces niche-specific disruptions in microbial diversity, taxonomic composition, and metabolic profiles, with the most severe microbial collapse observed in the endosphere. Functional prediction and network analysis revealed a transition from beneficial microbial processes toward saprotrophic and pathogenic activity, alongside the accumulation of antimicrobial metabolites. The integration of microbiome and metabolome datasets highlighted key microbe–metabolite associations and identified XTP and Gentamicin X2 as core metabolites mediating host–microbiome interactions. Our findings suggest that microbiome engineering strategies—such as the application of taxa (e.g., *Sphingomonas*, *Paenibacillus*) and metabolite-based priming (e.g., saikosaponin L, XTP)—hold promise for enhancing plant resistance against bacterial wilt. Future field-based research should focus on validating these candidate microbes and metabolites under diverse environmental conditions to assess their potential in sustainable disease management programs.

## Data Availability

The datasets presented in this study can be found in online repositories. The names of the repository/repositories and accession number(s) can be found below: https://www.ncbi.nlm.nih.gov/, PRJNA1223960 https://ngdc.cncb.ac.cn/?lang=en, PRJCA036159.

## Supplementary material

Supplementary Figure 1 Functional predictions of microbial communities in healthy and diseased plants across different root-associated niches using FAPROTAX. The left panel shows the relative abundance of functional groups in each condition, the middle panel illustrates the difference in proportions within a 95% confidence interval, and the right panel provides the corrected p-values for statistical significance. **(A)** Comparison of microbial functional composition between healthy (CK-S) and diseased (RS-S) plants in the root-surrounding soil. **(B)** Comparison between healthy (CK-R) and diseased (RS-R) plants in the rhizosphere. **(C)** Comparison between healthy (CK-E) and diseased (RS-E) plants in the endosphere.

Supplementary Figure 2 Taxonomic composition analysis of fungal communities at the phylum level, comparing healthy and diseased plant samples across root-surrounding soil **(A)**, rhizosphere **(B)**, and endosphere **(C)** niches.

Supplementary Figure 3 Alpha diversity analysis (Shannon and Chao1 indices) showing microbial richness and evenness differences between healthy and diseased plants across root-surrounding soil **(A)**, rhizosphere **(B)**, and endosphere **(C)** niches.

Supplementary Figure 4 Beta diversity analysis using Bray-Curtis distance-based Principal Coordinate Analysis (PCoA) across root-surrounding soil and rhizosphere **(A)**, and endosphere **(B)** niches.

Supplementary Figure 5 Top 10 differential fungal functional guilds between healthy (CK) and diseased (RS) plants across root-associated compartments. Boxplots show the relative abundance of the top 10 fungal functional guilds with statistically significant differences (FDR-adjusted p < 0.05) as predicted by FUNGuild. **(A)** Root-surrounding soil, **(B)** Rhizosphere and **(C)** Endosphere niches.

Supplementary Figure 6 Metabolite Profiling: PCA, Sample Correlation, and Clustering Analysis. **(A)** Principal Component Analysis (PCA) of metabolite profiles. **(B)** Sample correlation analysis based on Spearman Rank Correlation coefficients, used to evaluate biological replicate consistency. The x-axis and y-axis represent sample names, and the color intensity indicates the correlation coefficient (*r*). **(C)** Heatmap of metabolite clustering analysis, where the x-axis represents samples and the y-axis represents metabolites.

Supplementary Figure 7 KEGG pathway annotation of identified metabolites. KEGG database annotation was performed for all identified metabolites, selecting the top 20 KO pathway level 3 categories with the highest number of annotated metabolites. The grouped entries within the same box represent hierarchical classifications of KEGG pathways, corresponding to KO pathway level 2 and level 3. The bar length indicates the number of metabolites assigned to each pathway.

Supplementary Figure 8 Differential metabolites between healthy and diseased plants across root-associated niches. The figure presented the top 10 upregulated (red) and downregulated (green) metabolites in each comparison, based on log-transformed fold change (logFC). The bar labels indicate metabolite names, with bar length representing logFC values. **(A)** Comparison between the rhizosphere of healthy and diseased plants. **(B)** Comparison between the endosphere of healthy and diseased plants. **(C)** Comparison between the endosphere of diseased plants and the rhizosphere of diseased plants.

Supplementary Table 1 Summary of sequencing data quality and processing metrics, which presents sequencing data quality assessment across different root-associated niches (surrounding soil, rhizosphere, and endosphere) in healthy (CK) and diseased (RS) potato plants.

Supplementary Table 2 Species count at different taxonomic levels in root-associated niches. The species count is provided at multiple taxonomic levels, including kingdom, phylum, class, order, family, genus, and species.

Supplementary Table 3 Alpha diversity metrics of root-associated bacterial and fungal communities, including ASV/OUT count, ACE, Chao1, Simpson, Shannon, PD_whole_tree, and coverage, for bacterial and fungal communities across different root-associated niches (surrounding soil, rhizosphere, and endosphere) in healthy (CK) and diseased (RS) potato plants.

Supplementary Table 4 Topological characteristics of bacterial co-occurrence networks across root-associated niches.
