# Supplementary figures and images for: Bacterial wilt pathogen induced spatial shifts of root-associated microbiome and metabolome of potatoes
Source: Front Plant Sci. 2025 May 16;16:1577123. doi: 10.3389/fpls.2025.1577123 (PMC12122530; doi:10.3389/fpls.2025.1577123)

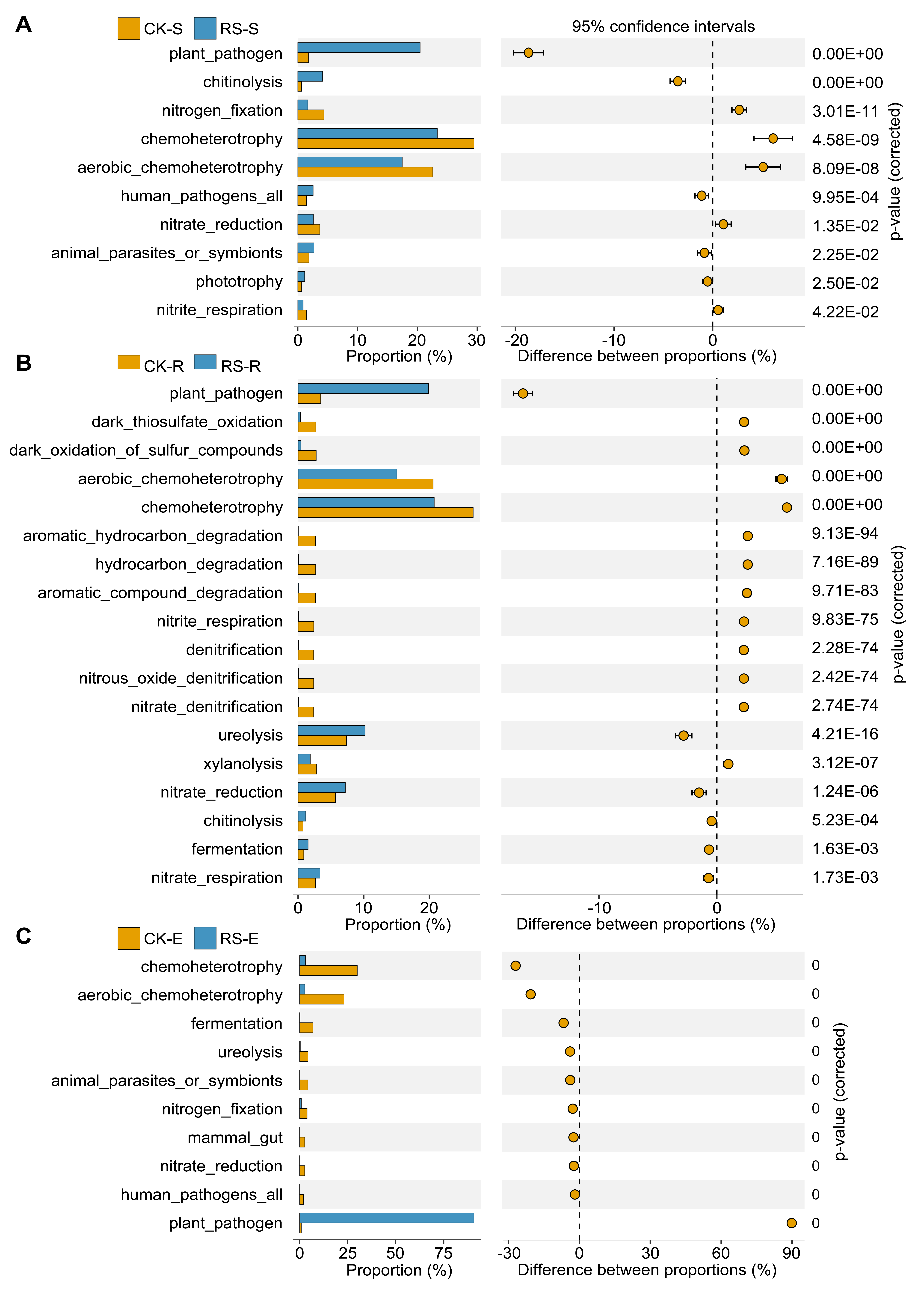

Supplement: Supplementary Figure 1 — Functional predictions of microbial communities in healthy and diseased plants across different root-associated niches using FAPROTAX. The left panel shows the relative abundance of functional groups in each condition, the middle panel illustrates the difference in proportions within a 95% confidence interval, and the right panel provides the corrected p-values for statistical significance. (A) Comparison of microbial functional composition between healthy (CK-S) and diseased (RS-S) plants in the root-surrounding soil. (B) Comparison between healthy (CK-R) and diseased (RS-R) plants in the rhizosphere. (C) Comparison between healthy (CK-E) and diseased (RS-E) plants in the endosphere. [file Image1.tif]

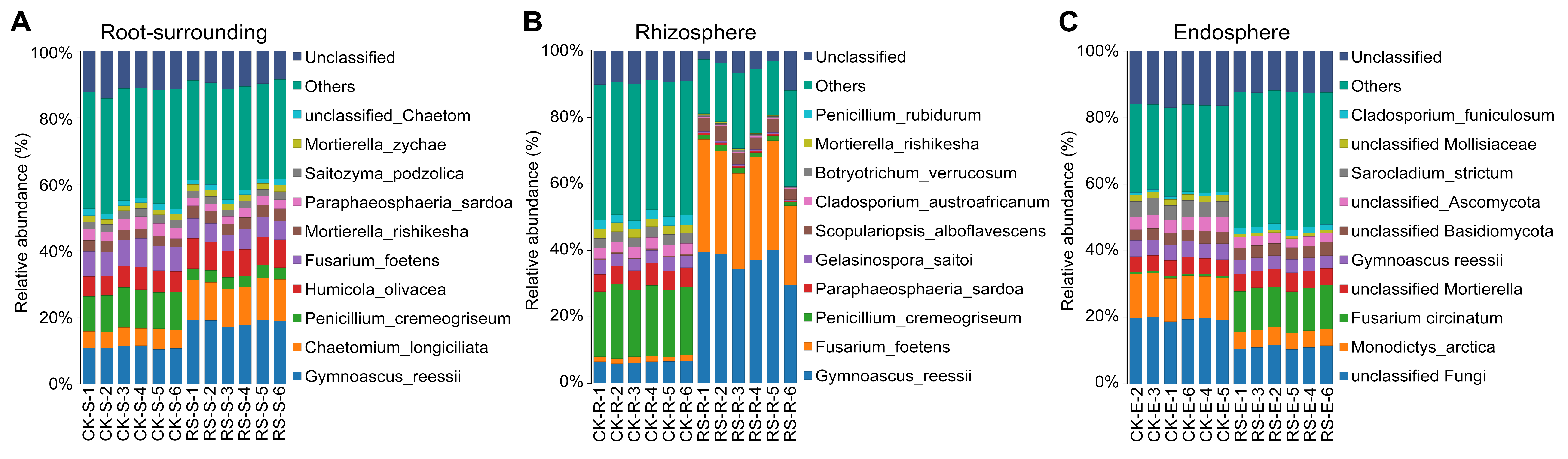

Supplement: Supplementary Figure 2 — Taxonomic composition analysis of fungal communities at the phylum level, comparing healthy and diseased plant samples across root-surrounding soil (A), rhizosphere (B), and endosphere (C) niches. [file Image2.tif]

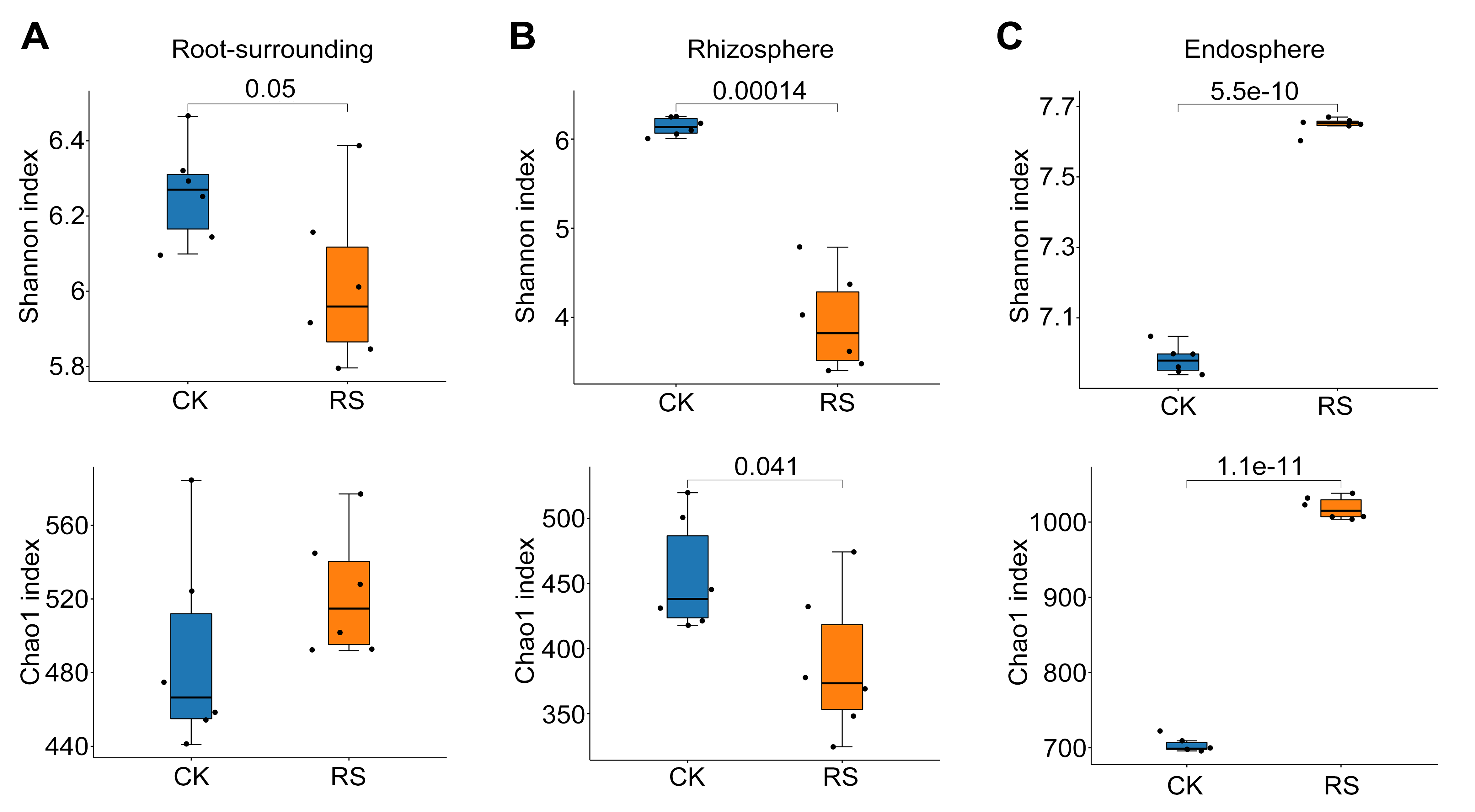

Supplement: Supplementary Figure 3 — Alpha diversity analysis (Shannon and Chao1 indices) showing microbial richness and evenness differences between healthy and diseased plants across root-surrounding soil (A), rhizosphere (B), and endosphere (C) niches. [file Image3.tif]

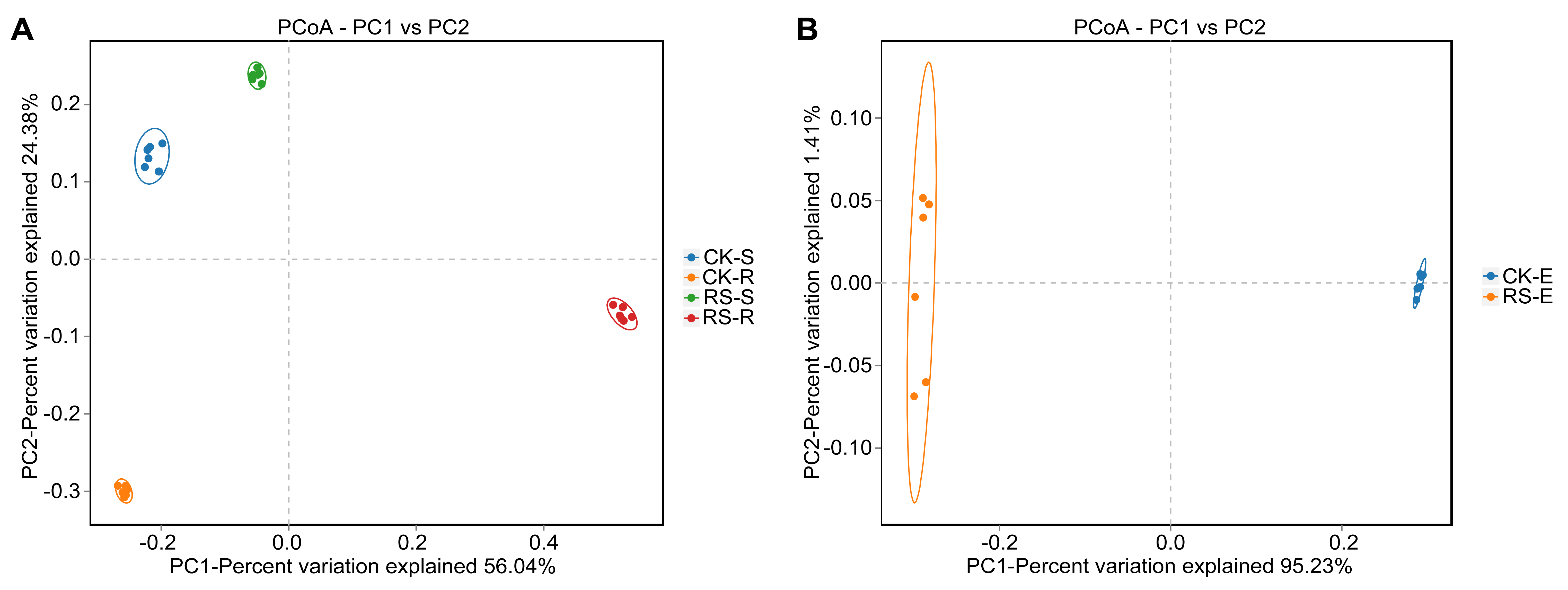

Supplement: Supplementary Figure 4 — Beta diversity analysis using Bray-Curtis distance-based Principal Coordinate Analysis (PCoA) across root-surrounding soil and rhizosphere (A), and endosphere (B) niches. [file Image4.tif]

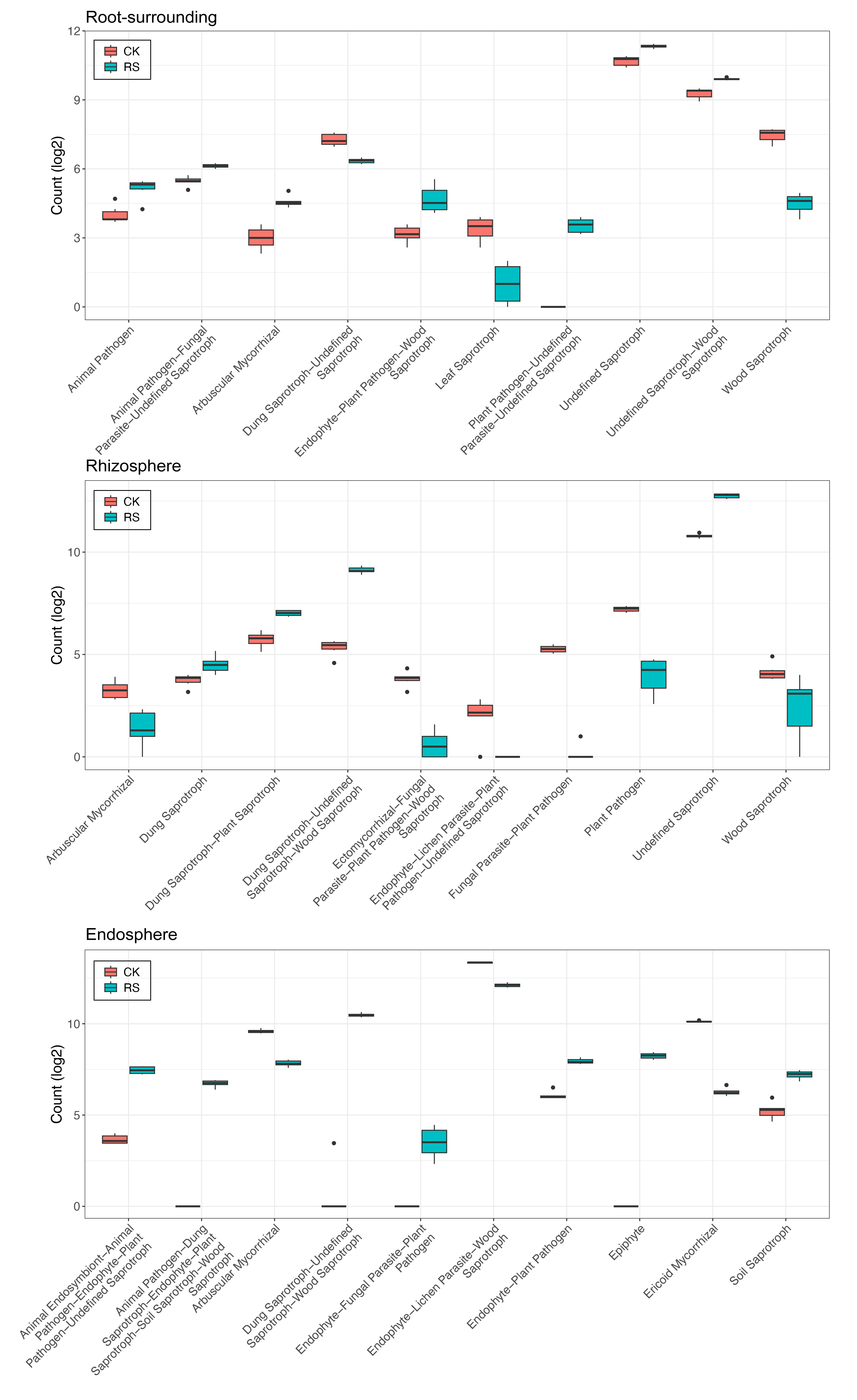

Supplement: Supplementary Figure 5 — Top 10 differential fungal functional guilds between healthy (CK) and diseased (RS) plants across root-associated compartments. Boxplots show the relative abundance of the top 10 fungal functional guilds with statistically significant differences (FDR-adjusted p < 0.05) as predicted by FUNGuild. (A) Root-surrounding soil, (B) Rhizosphere and (C) Endosphere niches. [file Image5.tif]

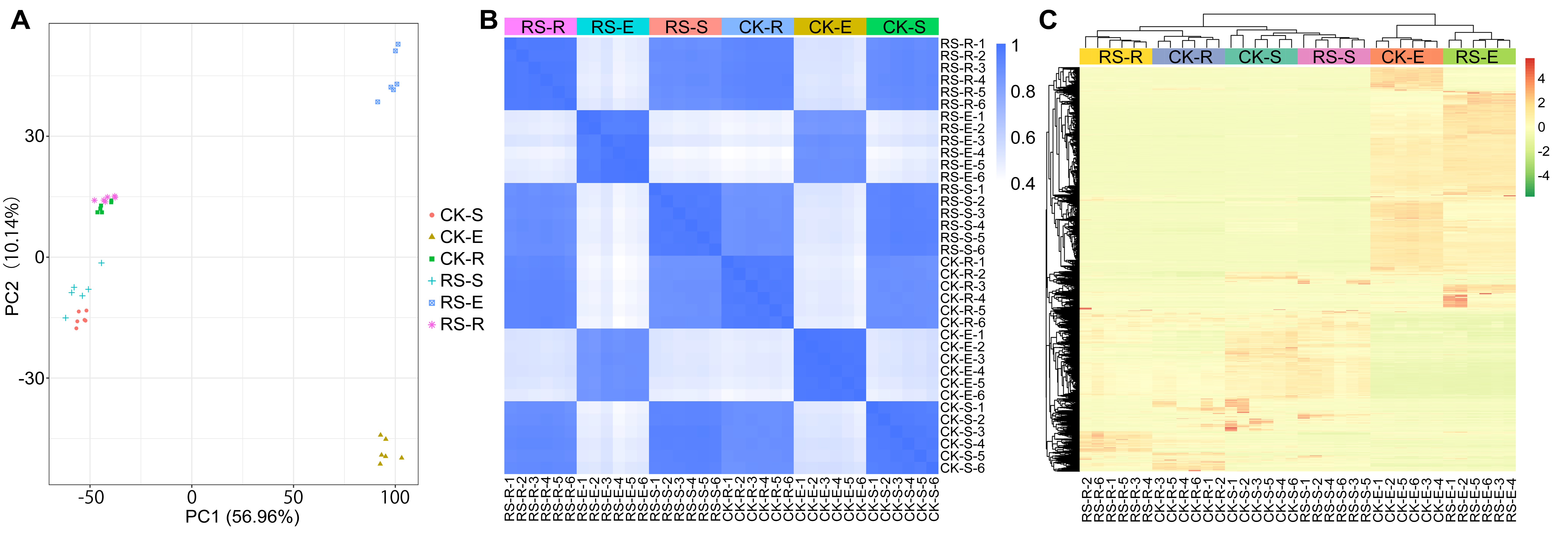

Supplement: Supplementary Figure 6 — Metabolite Profiling: PCA, Sample Correlation, and Clustering Analysis. (A) Principal Component Analysis (PCA) of metabolite profiles. (B) Sample correlation analysis based on Spearman Rank Correlation coefficients, used to evaluate biological replicate consistency. The x-axis and y-axis represent sample names, and the color intensity indicates the correlation coefficient (r). (C) Heatmap of metabolite clustering analysis, where the x-axis represents samples and the y-axis represents metabolites. [file Image6.tif]

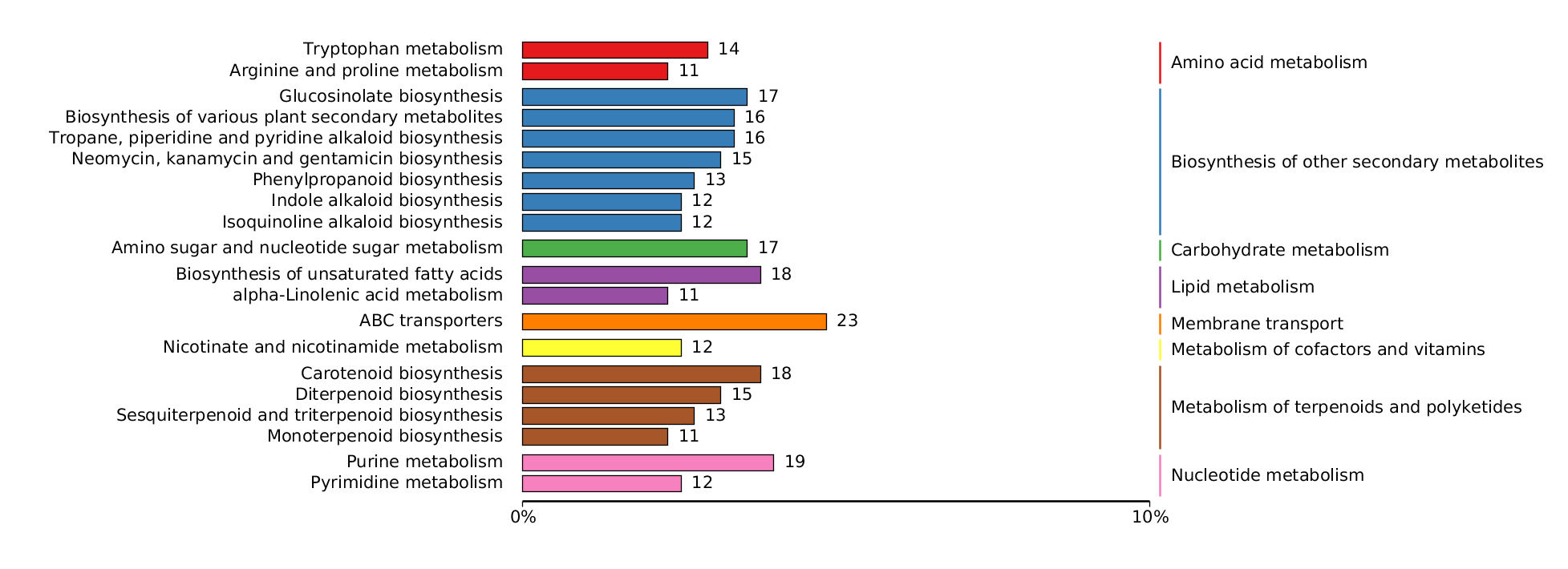

Supplement: Supplementary Figure 7 — KEGG pathway annotation of identified metabolites. KEGG database annotation was performed for all identified metabolites, selecting the top 20 KO pathway level 3 categories with the highest number of annotated metabolites. The grouped entries within the same box represent hierarchical classifications of KEGG pathways, corresponding to KO pathway level 2 and level 3. The bar length indicates the number of metabolites assigned to each pathway. [file Image7.tif]

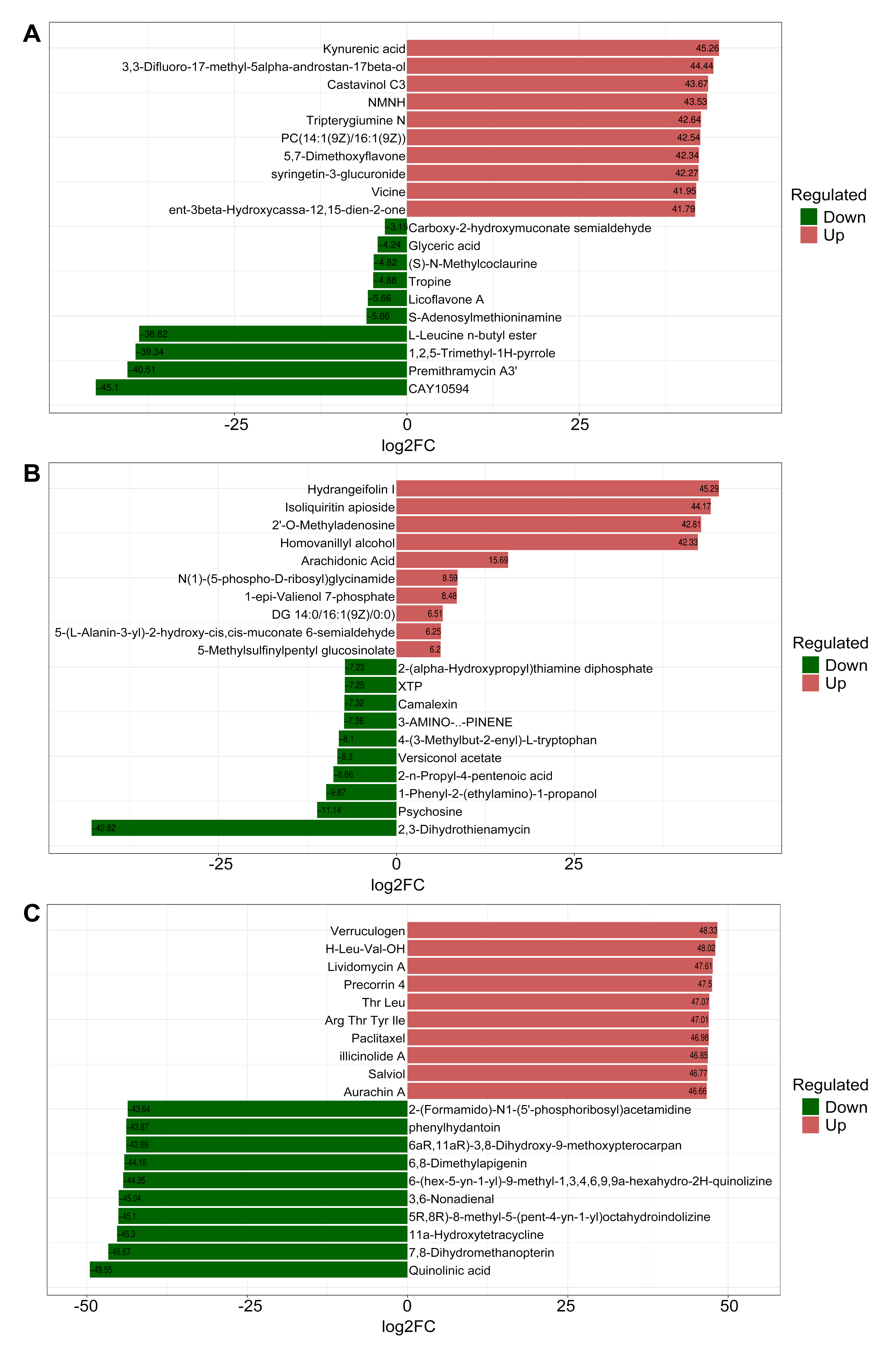

Supplement: Supplementary Figure 8 — Differential metabolites between healthy and diseased plants across root-associated niches. The figure presented the top 10 upregulated (red) and downregulated (green) metabolites in each comparison, based on log-transformed fold change (logFC). The bar labels indicate metabolite names, with bar length representing logFC values. (A) Comparison between the rhizosphere of healthy and diseased plants. (B) Comparison between the endosphere of healthy and diseased plants. (C) Comparison between the endosphere of diseased plants and the rhizosphere of diseased plants. [file Image8.tif]
